# Harmonization and qualification of intracellular cytokine staining to measure influenza-specific CD4^+^ T cell immunity within the FLUCOP consortium

**DOI:** 10.3389/fimmu.2022.982887

**Published:** 2022-10-20

**Authors:** Sarah Begue, Gwenn Waerlop, Bruno Salaun, Michel Janssens, Duncan Bellamy, Rebecca Jane Cox, Richard Davies, Elena Gianchecchi, Donata Medaglini, Emanuele Montomoli, Elena Pettini, Geert Leroux-Roels, Frédéric Clement, Anke Pagnon

**Affiliations:** ^1^ Research Global Immunology, Sanofi, Marcy L’Etoile, France; ^2^ Center for Vaccinology (CEVAC), Ghent University and University Hospital, Ghent, Belgium; ^3^ GlaxoSmithKline, Clinical Laboratory Sciences, Rixensart, Belgium; ^4^ The Jenner Institute Laboratories, University of Oxford, Oxford, United Kingdom; ^5^ Influenza Centre, Department of Clinical Science, University of Bergen, Bergen, Norway; ^6^ VisMederi, Siena, Italy; ^7^ Laboratory of Molecular Microbiology and Biotechnology (LA.M.M.B.), Department of Medical Biotechnologies, University of Siena, Siena, Italy; ^8^ Department of Molecular and Developmental Medicine, University of Siena, Siena, Italy

**Keywords:** cell-mediated immunity, FLUCOP, influenza vaccine, intracellular cytokine staining, polypositive T cells, standard operating procedure, qualification

## Abstract

Despite the knowledge that cell-mediated immunity (CMI) contributes to the reduction of severe influenza infection, transmission, and disease outcome, the correlates of protection for cell-mediated immunity remain still unclear. Therefore, measuring the magnitude and quality of influenza-specific T cell responses in a harmonized way is of utmost importance to improve characterisation of vaccine-induced immunity across different clinical trials. The present study, conducted as part of the FLUCOP project, describes the development of a consensus protocol for the intracellular cytokine staining (ICS) assay, in order to reduce inter-laboratory variability, and its qualification. In order to develop a consensus protocol, the study was divided into different stages. Firstly, two pilot studies evaluated critical parameters in the analytical (read-outs) and post-analytical (gating strategies and data analysis) methods applied by eight different laboratories within the FLUCOP consortium. The methods were then harmonized by fixing the critical parameters and the subsequent consensus protocol was then qualified by one FLUCOP member. The antigen-specific cell population was defined as polypositive CD4^+^ T cells (i.e. positive for at least two markers among CD40L/IFNγ/IL2/TNFα), which was shown to be the most sensitive and specific read-out. The qualification of this consensus protocol showed that the quantification of polypositive CD4^+^ T cells was precise, linear and accurate, and sensitive with a lower limit of quantification of 0.0335% antigen-specific polypositive CD4^+^ T cells. In conclusion, we provide the description of a harmonized ICS assay, which permits quantitative and qualitative evaluation of influenza vaccine-induced T cell responses. Application of this harmonized assay may allow for future comparisons of T cell responses to different influenza vaccines. It may facilitate future assessments of potential correlates of protection with the promise of application across other pathogens.

## 1 Introduction

Seasonal influenza causes almost 5 million serious cases per year and estimates of influenza-related mortality range between 290,000 to 645,000 deaths per year (1–3). Annual vaccination remains the most effective method of reducing morbidity and mortality associated with seasonal influenza. However, despite clinical evidence for the ability of influenza vaccines to protect against infection, currently hemagglutination-inhibition (HAI) antibody assays are the only accepted immune correlate of protection, and correlates of cellular protection induced by vaccines are not yet fully elucidated.

FLUCOP is a consortium of 22 members from eight European countries, encompassing academia, vaccine manufacturers, small- and medium-sized enterprise (SME)-based laboratories, and public health authorities, and is supported by the Innovative Medicines Initiative Joint Undertaking (IMI-JU) (4). The long-term goal of the FLUCOP project is the development and standardization of assays to assess influenza correlates of protection for use in future vaccine efficacy trials in order to facilitate the improvement of existing and development of novel influenza vaccines. This work is structured into a set of five work packages, with Work Package 2 focusing on the advanced understanding and application of cell-mediated immunity (CMI) in influenza vaccine development (4).

CMI is the immunity that is independent of antibodies but dependent primarily on the activation of antigen-specific T cells, with the subsequent release of various cytokines. Upon infection, CD8^+^ T cells are activated and can then differentiate into cytotoxic T lymphocytes which produce cytokines and effector molecules that kill virus-infected cells. Post-infection memory cells can remain in the blood allowing a quick response to secondary infection. Activated CD4^+^ T cells produce antiviral cytokines and support CD8^+^ T cell priming (5–7). Together T cells also support B cells in the production of antibodies. Given the importance of T cells in protective immunity to influenza virus, vaccination that can induce T cell responses are of interest. The use of flow cytometry and intracellular cytokine staining (ICS) assays allows for 1) the assessment of these cytokine-producing cells and has been shown to quantitatively and qualitatively evaluate vaccine-induced immunogenicity, and for 2) analysis of immune correlates of protection (8). However, due to the many variations in assay parameters and variations in the analysis of the data acquired through flow cytometry, the ICS assay remains variable when comparing data generated across laboratories (9–11). Guidelines suggest that CMI analyses can provide supportive information for the evaluation of influenza vaccine immunogenicity (12). However, in order for the assay to be used optimally in clinical trials, the assay must be qualified or validated (9, 13). Harmonization and standardization of this method allows for the definition of a range of responses within which the assay is controlled, allowing for the comparison of CMI data generated by different laboratories.

The aim of this study was to harmonize the ICS assay for use in evaluating seasonal human influenza vaccines. We performed two pilot studies to determine the optimal protocol for the analytical and post-analytical phases, followed by the qualification of the harmonized ICS assay.

## 2 Materials and methods

This study consisted of three parts: pilot study 1 theoretically evaluated and practically assessed in-house SOPs across different consortium laboratories in order to develop a standardized SOP; pilot study 2 evaluated only post-analytical procedures across laboratories in order to develop guidelines to harmonize post-analytical procedures; and the final part of the study was the qualification of the harmonized ICS SOP.

### 2.1 Samples

Peripheral blood mononuclear cells (PBMC) were isolated from buffy coats obtained from healthy blood donors (Red Cross Flanders). PBMC were also isolated from blood sampled from healthy volunteers that participated in a clinical vaccine trial that was carried out specifically for these studies. For this, venous blood was collected in heparin-coated blood collection tubes prior to and 7 days after the administration of a seasonal influenza vaccine (alfa-RIX-Tetra^©^ [season 2015/2016 or 2016/2017]). Ethical approvals for this study and the use of blood collected from Red Cross donors were given by the Ethical Committee of the Ghent University Hospital.

PBMC were isolated according to the standardized procedure FLUCOP SOP for PBMC isolation and cryopreservation, (**Appendix 1**). In brief, venous blood samples were diluted 1:2 in Hanks buffered salt solution (HBSS), and buffy coats were brought to a total volume of 300 mL in HBSS. PBMC were isolated by isopycnic centrifugation using Lymphoprep™. Subsequently, cells were washed twice in HBSS, suspended in freezing medium (10% dimethyl sulfoxide [DMSO]/90% fetal bovine serum [FBS]), frozen at a concentration of ≥ 5 to ≤ 20 million cells/mL within 24h (buffy coats) or 6h (whole venous blood samples) after blood collection and finally stored in the vapor or liquid-phase of liquid nitrogen until use.

All cryovials were identified with unique codes without any reference to their source. All samples later distributed to other laboratories were selected from this PBMC biobank based on their pre-examined CMI immune responses against influenza antigens.

### 2.2 Pilot study 1

Each participating laboratory was provided with the same selection of 24 cryopreserved PBMC samples. The laboratories were blinded to the samples. Each laboratory was also provided with antigens (recombinant hemagglutinin (HA) of H1 A/California/07/2009 and B/Phuket/3073/2013, Protein Sciences, provided by Sanofi) for stimulation at a final concentration of 0.25 µg/mL. The laboratories were instructed to select the immunological marker(s) they normally use for their ICS assay. The laboratories then tested each of the 24 samples using their in-house SOP, and reported the parameters of their procedure and the results of their analysis (**Supplementary Table 1**).

The collected data were analyzed in order to address the questions ‘What markers, or combination of markers, lead to a correct classification/ranking of the samples?’ and ‘What is the impact of the addition or absence of markers on the sensitivity of the assay?’. The reported results were expressed as a percentage of the parent population and used after background subtraction in further analyses, and expressed per sample and per marker. A mean response for the 24 samples per type of response and laboratory was calculated, providing an indication of the sensitivity of the methods to detect the CD4 response. As expected, no CD8 responses were detected after stimulation with a recombinant protein and therefore this response was not further investigated. The captured parameters were compared with the data showing the highest levels of variation. Those parameters considered to greatly induce variation were harmonized. Less critical parameters were not fixed to allow for sufficient flexibility for the laboratory. A consensus protocol, agreed upon by all Work Package 2 partners, was established.

### 2.3 Pilot study 2

Each participating laboratory was provided with a set of 20 (10 paired background and stimulated) blinded, anonymized, non-gated and not compensated flow cytometry standard (FCS) files. The provided datasets were FCS 2.0, which should be readable by all software packages used for cytometry data analysis. The staining included in the samples is provided in **Supplementary Table 2**. The participants were asked to process the FCS files by applying their in-house procedure and to report the obtained results together with a pdf of the gating strategy applied. For result reporting, two specific instructions were given, 1) Enumerate antigen (Ag)-specific living or viable IFNγ-producing CD4^+^ or CD8^+^ T cells and extrapolate per million parent CD4^+^ or CD8^+^ T cells and 2) Enumerate Ag-specific living or viable CD4^+^ or CD8^+^ T cells producing at least 2 of the tested markers and extrapolate per million parent cells.

The results obtained by each laboratory were tabulated and descriptive statistics were performed. We then looked for the common denominators and how this correlated with the level of quality observed in the pdf files. The gating strategy was based on discussion and consensus by all participating laboratories, using the laboratories with the highest antigen-specific responses as examples.

The consensus protocol developed following these discussions can be found in **Appendix 2**.

### 2.4 ICS qualification assay

#### 2.4.1 Standard operating procedure (SOP)

The qualification of the consensus SOP for the ICS assay was done by experienced technicians at Sanofi (Marcy L’Etoile, France) using 212 frozen PBMC cryovials from 26 subjects from a characterized biobank (provided by Ghent University). Briefly, PBMC samples were thawed rapidly at 37°C, then washed and suspended in culture medium ready for stimulation. Cell count and cell viability were determined. Prior to *in vitro* cell stimulation, cells were suspended in AIM-V medium (Gibco) at a concentration of 10x10^6^/mL. One million PBMCs per well were stimulated with 2 influenza monovalent strains from the Vaxigrip vaccine, A/California/07/2009 (Sanofi) at 1µg/mL or B/Phuket/3073/2013 (Sanofi) at 2µg/mL, or Tetanus Anatoxin (ATT) used at the final concentration of 5µg/mL (Sanofi), or with medium alone as the negative control. Anti-human CD28 (Biolegend) at 1µg/mL and anti-human CD49d (Biolegend) at 1µg/mL were also added as co-stimulators to all wells. Cells were incubated at 37°C and 5% CO_2_ for 2 hours followed by 16 hours in the presence of 10µg/mL Brefeldin A (BFA; Sigma).

The staining procedure and materials are shown in **Supplementary Table 3**. Following stimulation, cells were incubated with EDTA at 2mM/well for 15 minutes at room temperature, then washed with PBS. Viability was assessed with Live/Dead Fixable Aqua and cell-surface staining was done with anti-human CD4 and anti-human CD8 antibodies for 20 minutes at room temperature. Cells were then permeabilized and fixed with Cytofix/Cytoperm (BD Pharmingen) for 20 minutes at room temperature and washed twice with Perm/Wash (BD Pharmingen) buffer. Intracellular staining was done with anti-human CD3, anti-human TNFα, anti-human IL2, anti-human CD40L and anti-human IFNγ antibodies for 20 minutes. Plates were then kept at 5°C and protected from light until acquisition, which was done within 24 hours.

Samples were acquired on a BD LSR II flow cytometer (BD Biosciences), instrument configuration shown in **Supplementary Table 4**, with samples collected from a 96-well plate using high throughput sample (HTS) device (BD Biosciences). PMT voltages were set using unstained cells, the matrix compensation was calculated using beads; with targeting a collection of 100,000 to 200,000 CD3^+^CD4^+^ gated events in each well for this qualification of the harmonized methods.

Using Flow Jo 10.3 (BD Biosciences), gates for IFNγ, TNFα, IL-2 and CD40L expression cells were created on CD4^+^ gate; Boolean gating was then performed resulting in 16 populations. An example of the applied gating strategy is shown in **Supplementary Figure 1**. The cell counts of the 11 different populations expressing at least two of the markers were summed, divided by the number of CD4^+^ cells acquired in each sample and multiplied by 100, this was then corrected by subtracting the corresponding percentage of polypositive cells in the unstimulated cell culture (background). This provides the percentage of antigen-specific polypositive CD4^+^ T cells.

The SOP used for the qualification of the method differed slightly from the consensus protocol in **Appendix 2**: PBMCs were stimulated in AIM-V medium (Gibco) instead of complete RPMI containing 10% FBS, PBMCs were stimulated in the presence of BFA for 16 hours instead of 18 hours, after staining samples were incubated for 15 minutes in 2mM EDTA, the Live/Dead staining was done separately (before the staining of the extracellular markers), staining steps were done at 5°C instead of RT, washing buffer was PBS with 0.5% BSA instead of 1% FCS, washing steps were performed with 200µL and fixation was performed using 100µL Cytofix/CytoPerm for 15 minutes at 5°C instead of 20 minutes at RT.

#### 2.4.2 Data analysis

The qualification assays were performed on the CD4^+^ T cell responses, as no CD8^+^ T cell responses could be detected. Five parameters were assessed for the ICS assay qualification: the limit of blank (LOB), precision (intra- and inter-assay and lower limit of intermediate precision [LLOIP]), linearity (including relative accuracy and limit of linearity [LOL]), lower limit of quantification (LLOQ) and specificity.

#### 2.4.3 Limit of blank

The LOB is the assay background, meaning the highest response expected from negative/naive samples. To determine the LOB, the negative controls of the eight samples quantified in quadruplicate in four runs from the precision study were used. Intra-run duplicates of “delta background” (negative control 1 minus negative control 3 and negative control 2 minus negative control 4) were calculated, providing 64 values of delta background. The LOB was set at the 95% of the distribution of the absolute values of the delta background.

#### 2.4.4 Precision

Precision expresses the agreement between a series of measurements obtained from multiple results from the same homogeneous sample generated by the test procedure. Intermediate or inter-assay precision expresses the variation between days but within laboratory. Precision was considered at 2 levels: repeatability and intermediate precision. Precision was tested using eight samples, two high, two medium and four low responders, stimulated with either A/California/07/2009, or B/Phuket/3073/2013. Intra-assay precision, or repeatability, was determined with each stimulation tested four times within a single run, on the same day by the same operator. Inter-assay, or intermediate, precision was estimated by testing the same eight samples in four independent runs, by two different operators, on different days. For repeatability and intermediate precision, results were background (medium) corrected and reported as % polypositive CD4^+^ T cells. The results were log_10_ transformed and the mean frequencies calculated for each sample. The percentage of geometric coefficient of variation (%GCV) was used to assess precision. The LLOIP is the lowest value observed of one sample for which the GCV was ≤40%.

#### 2.4.5 Linearity and relative accuracy

Linearity is the ability of the analytical procedure to elicit test results that are directly proportional to the frequency of live CD4^+^ T cells in the sample. In the linearity experiment, three samples were tested, one high, one medium and one low responder, and for each sample four independent stimulations with A/California/07/2009 were performed in a single run. Linearity was determined by performing independent dilutions of the stimulated samples with non-stimulated autologous PBMCs, with at least six dilutions per sample. The expected results were calculated by multiplying the value from the undiluted sample by the dilution factor, for each replicate, and then subtracting the geometric mean of the unstimulated sample. The same geometric mean of the unstimulated sample was subtracted from each observed value to obtain the background corrected observed value. The results were log_10_ transformed and linearity was assessed by regressing the observed corrected against the expected corrected mean. Linearity was demonstrated when the 90% CI of the slope was included in the equivalence interval of 0.80 to 1.25. The relative accuracy, which demonstrated the closeness between the expected and observed values, was assessed by calculating the CI of the mean percent recovery calculated as: (individual observed corrected/expected corrected geometric means) and was demonstrated if the 90% CI of the mean recovery percentage was in the equivalence interval of 50% to 200%. The LLOL is the lowest value of the linearity and accuracy domain.

#### 2.4.6 Lower limit of quantification (LLOQ)

The LLOQ was determined from the linearity and precision data and was defined as the highest value observed between the LLOL (see *Linearity and relative accuracy*) and LLOIP (see *Precision*).

#### 2.4.7 Specificity

Specificity is the ability of the assay to assess unequivocally the analyte in the presence of components that may be expected to be present in the sample matrix. The specificity of the ICS assay was determined in 25 paired samples that were collected pre- and 7 days post-vaccination. These paired samples were tested in two different runs and were stimulated with an influenza-specific antigen (A/California) or an irrelevant antigen (ATT) to examine the specificity of the assay. This would provide a single, background (medium)-subtracted result per timepoint which was reported as % corrected polypositive CD4^+^ T cells. The paired data, pre- and post-vaccination, was compared using a Wilcoxon signed-rank test, and for specificity for inducing a CD4^+^ T cell response, the influenza antigen stimulation should demonstrate a significant difference between pre- and post-vaccination results (p<0.05), when there should be no difference for the ATT stimulation (p≥0.05).

All analyses were done using SAS v9.4.

## 3 Results

### 3.1 Pilot study 1

**Figure 1:**
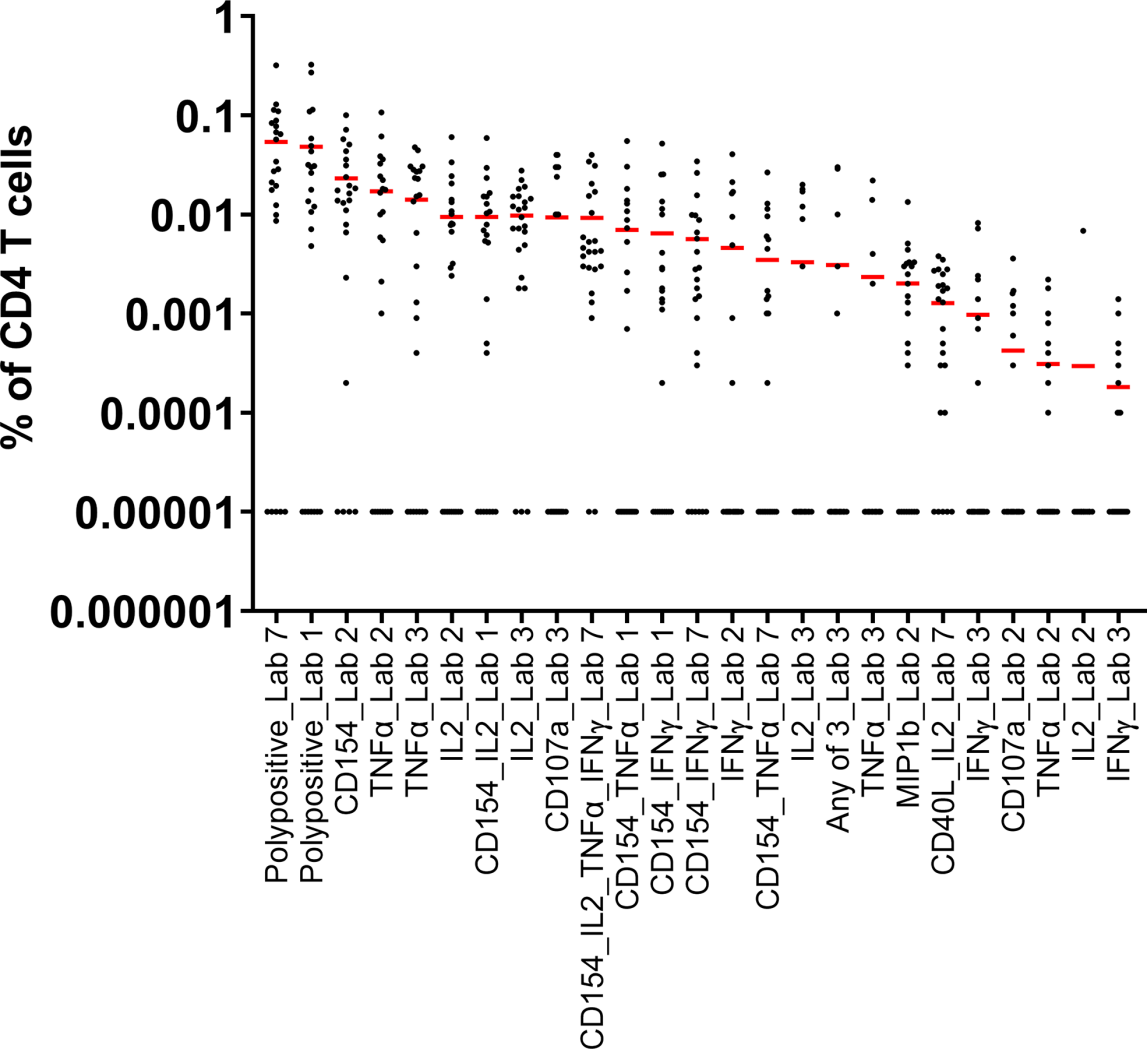
Pilot Study 1: Evaluation of data from each of the participating laboratories. Each dot represents the A/California/07/2009 stimulated and background subtracted % of parent cells for a single sample. The red line indicates the mean of the 24 samples for each outcome for each laboratory. All 0 values were replaced by 0.00001 in order to represent the data. Seven laboratories participated in pilot study 1, however one laboratory did not provide data in time for inclusion in the assessment of the results. IFN, interferon; IL, interleukin; TNF, tumor necrosis factor.

Seven laboratories participated in pilot study 1. The information provided by the laboratories on their in-house parameters for ICS demonstrated a wide variation in processes, in the assay panels used, and in the types of reported responses (**Table 1**). The mean responses per CD4^+^ subpopulation and laboratory indicated that the measurement of polypositivity appeared to be the less variable and more specific ICS method (**Figure 1**). The added value of other markers remains unclear. As expected, using samples from subjects vaccinated with a protein vaccine and restimulating the cells with a recombinant protein, CD8^+^ T cell responses were rarely detected and therefore not reported. The harmonization of methodology is needed to develop a consistency of results. Following discussion, a consensus was reached between the participating FLUCOP partners on a set of identified parameters that can induce assay variation (**Table 2**), but still allowed for the use of different stimulating antigens and different fluorochromes depending on the availability or configuration of the flow cytometer.

**Table 1:** Pilot Study 1: Information about the in-house SOP for each participating laboratory.

Assay steps	Laboratories						
	1	2	3	4	5	6	7
**Thawing Temperature**	37°C	37°C	37°C	37°C	37°C	37°C	37°C
**Cell Count Method**	Automated	Automated	Automated	Automated	Manually	Automated	Automated
**Viability Pre-stimulation**	PI	Viacount assay (Luminex)	Trypan Blue	CASY Model TT	Trypan Blue	CTL-LDC Kit	PI
**Viability as part of flow cytometry panel**	Live/Dead	Live/Dead	Live/Dead	Live/Dead	Live/Dead	Live/Dead	Live/Dead
**Cell number/well (during stimulation)**	1,000,000	1,000,000	1,000,000	1,000,000	2,000,000	600,000-1,200,000	1,000,000
**Resting**	No	overnight (17h)	overnight (18h)	8h	3h	overnight	No
**Stimulation duration without secretion inhibitor**	2 h	3 h	2 h	0 h	2 h	2h	2 h
**Stimulation duration with secretion inhibitor**	16 h	14 h	16 h	8 h	16 h	14 h	16 h
**Secretion inhibitor**	BFA	BFA/Monensin	BFA	BFA/Monensin	BFA/Monensin	BFA	BFA
**Costimulation**	Anti-CD28/CD49d	No	Anti-CD28/CD49d	Anti-CD28/CD49d	Anti-CD28/CD49d	Anti-CD28/CD49d	Anti-CD28/CD49d
**Extracellular Panel**	L/D, CD4/CD8	L/D, CD14/CD3/CD4/CD8/CD197/CD45RA	L/D	L/D, CD3/CD4/CD8/CD19	L/D	L/D, CD4/CD8	L/D, CD4/CD8
**Intracellular Panel**	CD3/CD40L/IFNγ/IL2/TNFα	CD107a/IFNγ/IL2/CD40L/TNFα/MIP1b	CD3/CD4/CD8/CD40L/IFNγ/IL2/TNFα	IFNγ/IL2/TNFα	CD3/CD4/CD14/CD19/CD8/IFNγ/IL2/TNFα/CD107a	CD3/CD40L/IFNγ/IL2/TNFα	CD3/CD40L/IFNγ/IL2/TNFα

BFA, Brefeldin A; IFN, interferon; IL, interleukin; L/D, live/dead; PI, propidium iodide; SOP, standard operating procedure; TNF, tumor necrosis factor.

Seven laboratories participated in pilot study 1, however one laboratory did not provide data in time for inclusion in the assessment of the results.

**Table 2:** Parameters selected for the ICS consensus assay.

Assay step	Recommendations
**Thawing Temperature**	37°C
**Cell Count Method**	Automated
**Viability method Pre-culture**	PI/Trypan Blue/Other
**Viability Post-Culture**	Live/Dead
**Cell number/well (during stimulation)**	1,000,000
**Overnight Resting**	None
**Extracellular Panel**	Live/Dead staining, CD4/CD8
**Intracellular Panel**	CD3/CD40L/IFNγ/IL2/TNFα
**Read-Out**	Polypositive CD3^+^/CD4^+^ or CD3^+^/CD8+ polypositive cells (CD40L, IFNγ, IL2, TNFα)
**Minimal number of acquired cells**	Acquire at least 75,000 Live CD3+CD4+ parent cells Optimally 100,000 CD4+ T cells should be acquired

ICS, intracellular cell staining; IFN, interferon; IL, interleukin; PI, propidium iodide; TNF, tumor necrosis factor.

### 3.2 Pilot study 2

**Figure 2:**
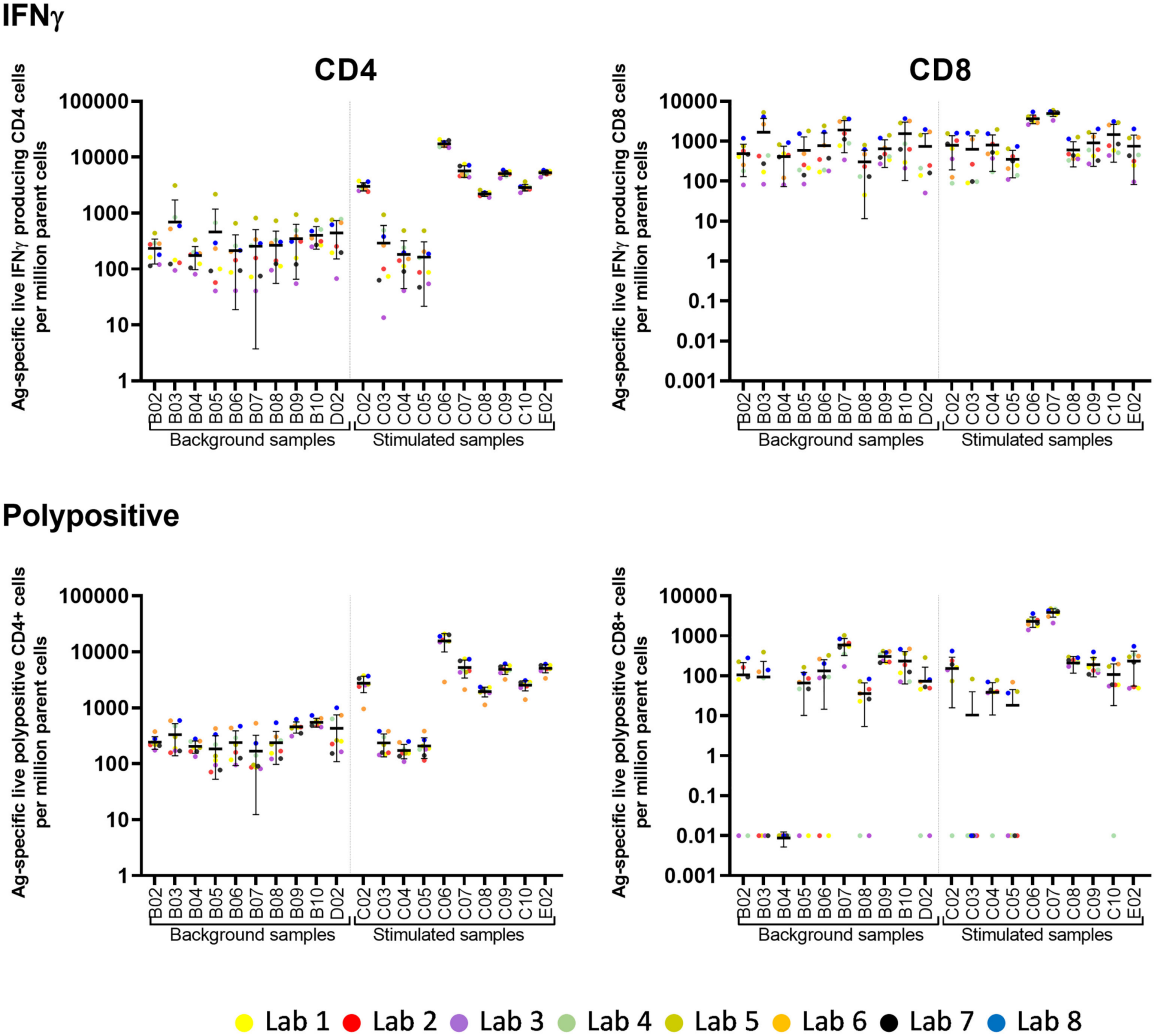
Pilot Study 2: Variability of post-analytical analysis between laboratories. Eight laboratories participated in pilot study 2. Each laboratory analyzed fcs files from 10 unstimulated (background) and 10 stimulated samples (x-axis). Each dot represents the number of Ag-specific live IFNγ or polypositive CD4^+^ or CD8^+^ cells per million parent cells from one laboratory. The horizontal line with error bars indicate the mean +/- standard deviation per sample. IFN, interferon; PP, polypositive.

**Figure 3:**
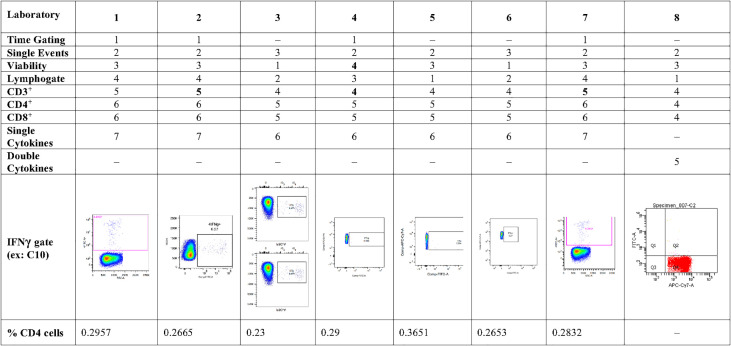
Pilot Study 2: Gating practices for cytokine secreting CD4^+^ T cells from each of the participating laboratories, and examples of a single cytokine gate (IFNγ) from each of the laboratories Eight laboratories participated in pilot study 2-, gating strategy did not show single IFNγ-secreting cells; IFN, interferon.

Eight laboratories participated in pilot study 2 and provided data of their analysis of the flow cytometry data for IFNγ and polypositive CD4^+^ and CD8^+^ T cells (**Figure 2**). The variability of analytical results is higher in unstimulated (background) samples than in stimulated samples, and in CD8^+^ cells than in CD4^+^ cells, as these responses are at the lower end of the analytical range. The results are less variable, especially for the unstimulated samples, if gated on polypositive cells instead of only IFNγ positive cells, presumably increasing specificity and sensitivity (**Figure 2**). **Figure 3** shows the gating sequence employed by each laboratory and the variability of the IFN-γ^+^ gate for a randomly selected sample (sample C10). **Supplementary Figure 2** shows the complete gating strategy per laboratory for another randomly selected sample (sample C05). The gate sequence was very different between laboratories as shown for example for CD3^+^ cells which had an impact on the outcome of the analysis. Additionally, even when laboratories applied the same gate sequence, the positioning of the gates had an important impact on the results (**Supplementary Figure 2** **)**. However, if gates were placed in the same order and at comparable positions, as seen for laboratories 1, 2 and 7 (**Supplementary Figure 2**), the variability in results was substantially reduced. A consensus was reached regarding gating practices for ICS that encompassed applying time-gating (selecting a stable signal over time), excluding double-events using a FSC-A/FSC-H gate, using density (or zebra) plots, applying bi-exponential gating with care, setting gates as such to avoid losing polypositivity by CD3^+^ internalization, and using back-gating to check gating strategy on high positive cells.

After the two pilot studies were completed, a harmonized SOP was generated based upon the critical parameters upon which the members of Work Package 2 agreed. This SOP was then qualified by one member.

### 3.3 ICS assay qualification

#### 3.3.1 Limit of blank

The LOB, based on the 95^th^ percentile of the distribution (data not shown) of the absolute values of the delta background, was 0.0113% polypositive CD4^+^ T cells (**Table 3**).

#### 3.3.2 Precision

**Figure 4:**
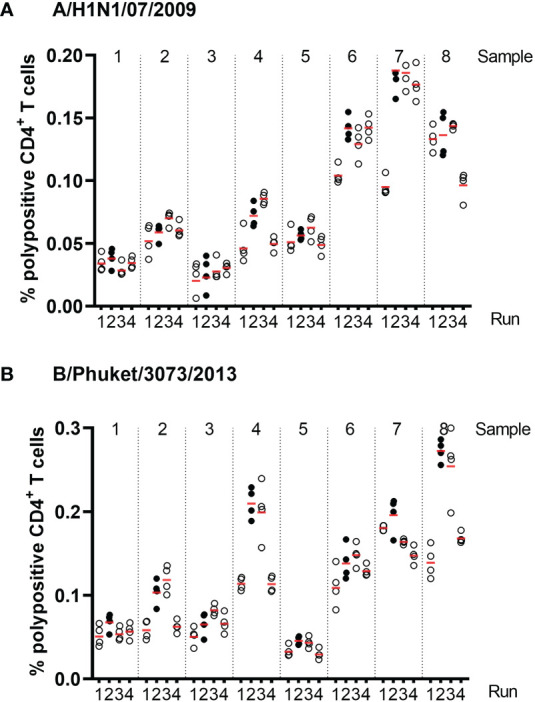
Frequencies of corrected A/California/07/2009-specific **(A)** and B/Phuket/3073/2013-specific **(B)** polypositive CD4^+^ T cells in eight different samples, from four different test runs by two different operators in assays of intra- and inter-assay precision. Eight different samples were tested, 2 high, 2 medium and 4 low responders. Each dot represents an individual background-corrected response reported as a percent of polypositive CD4^+^ T cells. The horizontal lines demonstrate the mean response for each sample per run. Each color represents a different operator.

**Figure 5:**
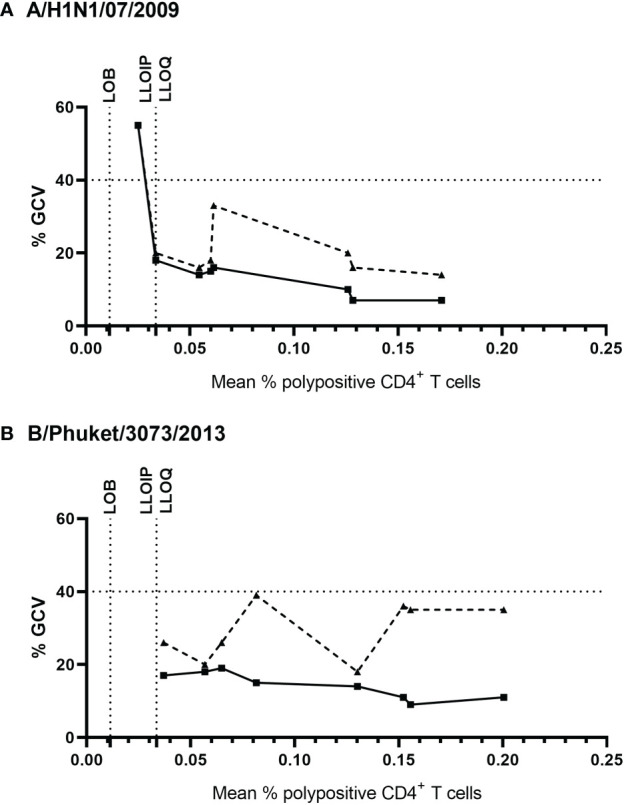
Intra- and inter-assay %GCV for A/California/07/2009 **(A)** and B/Phuket/3073/2013 **(B)**. The solid black line indicates the intra-assay %GCV, the dashed line indicates the inter-assay %GCV. For each of the 8 samples tested for precision, the %GCV was calculated (y-axis) and is plotted against the mean response (x-axis). The horizontal dotted line represents a GCV of 40%, the vertical dotted lines indicate the LOB, and LLOIP/LLOQ. %GCV, percentage of geometric coefficient of variation; LLOIP, lower limit of intermediate precision; LLOQ, lower limit of quantification; LOB, limit of blank.

Precision was assessed using eight PBMC samples, which were tested four times within each of four assay runs with two operators. **Figure 4** shows the % of corrected A/California/07/2009-specific (upper panel) and B/Phuket/3073/2013-specific (lower panel) polypositive CD4^+^ T cells (**Figure 4**). The %GCV of the intra- and inter-assay precision was calculated for each sample and for each stimulation, and the mean response per sample was plotted against its %GCV for A/California/07/2009 (**Figure 5A**) and B/Phuket/3073/2013 (**Figure 5B**). For A/California/07/2009 the %GCV varied from 7–55% for repeatability and 14–55% for intermediate precision and had the tendency to decrease with increasing mean response. For B/Phuket/3073/2013, the %GCV varied from 9–19% for repeatability and 18–39% for intermediate precision and was rather constant regarding the mean response. The %GCV for all samples and all stimulations combined was determined as 23% for intra-assay precision and 28% for inter-assay precision, corresponding to a 1.6-fold and 1.7-fold difference between two values, respectively (**Tables 3**,**4**). Therefore, this consensus method is considered precise. The LLOIP was 0.0335% polypositive CD4^+^ T cells, being the lowest value with a GCV ≤40% (**Table 3**).

**Table 3:** Summary table for qualification assay results.

Test	Qualification parameter	Result
	LOB	0.0113%
**Precision**	Intra-assay (%GCV ≤40%)	23% (19% B/Phuket, 26% A/California)
	Inter-assay (%GCV ≤40%)	28% (27% B/Phuket, 28% A/California)
	LLOIP	0.0335%
**Linearity**	Range	0.0105% to 0.2208%
	LLOL	0.0105%
	Slope (90% CI within 0.80, 1.25)	0.935 (0.90, 0.97)
	**LLOQ** (Highest value between the LLOL and the LLOIP)	0.0335%
**Specificity**	Pre- and post-vaccination difference	A/California; p<0.0001
	Pre- and post-vaccination difference	ATT, p=0.0637

LLOL, lower limit of linearity; LLOIP, lower limit of intermediate precision; LOB, limit of blank; %GCV, percentage of geometric coefficient of variation.

**Table 4:** Precision results for eight samples for each stimulation (A/California/07/2009 and B/Phuket/3073/2013).

Sample	Stimulation	Variance		Precision		%GCV	
		Repeatability	Intermediate precision	Repeatability	Intermediate precision	Repeatability	Intermediate precision
**All samples**	**A/California/07/2009**	0.01214	0.01462	x/1.7	x/1.7	26%	28%
	**B/Phuket/3073/2013**	0.00644	0.01372	x/1.4	x/1.7	19%	27%
**All samples**	**All stimulations**	0.00957	0.0141	x//1.6	x/1.7	23%	28%

%GCV, percentage of geometric coefficient of variation.

#### 3.3.3 Linearity and relative accuracy

**Figure 6:**
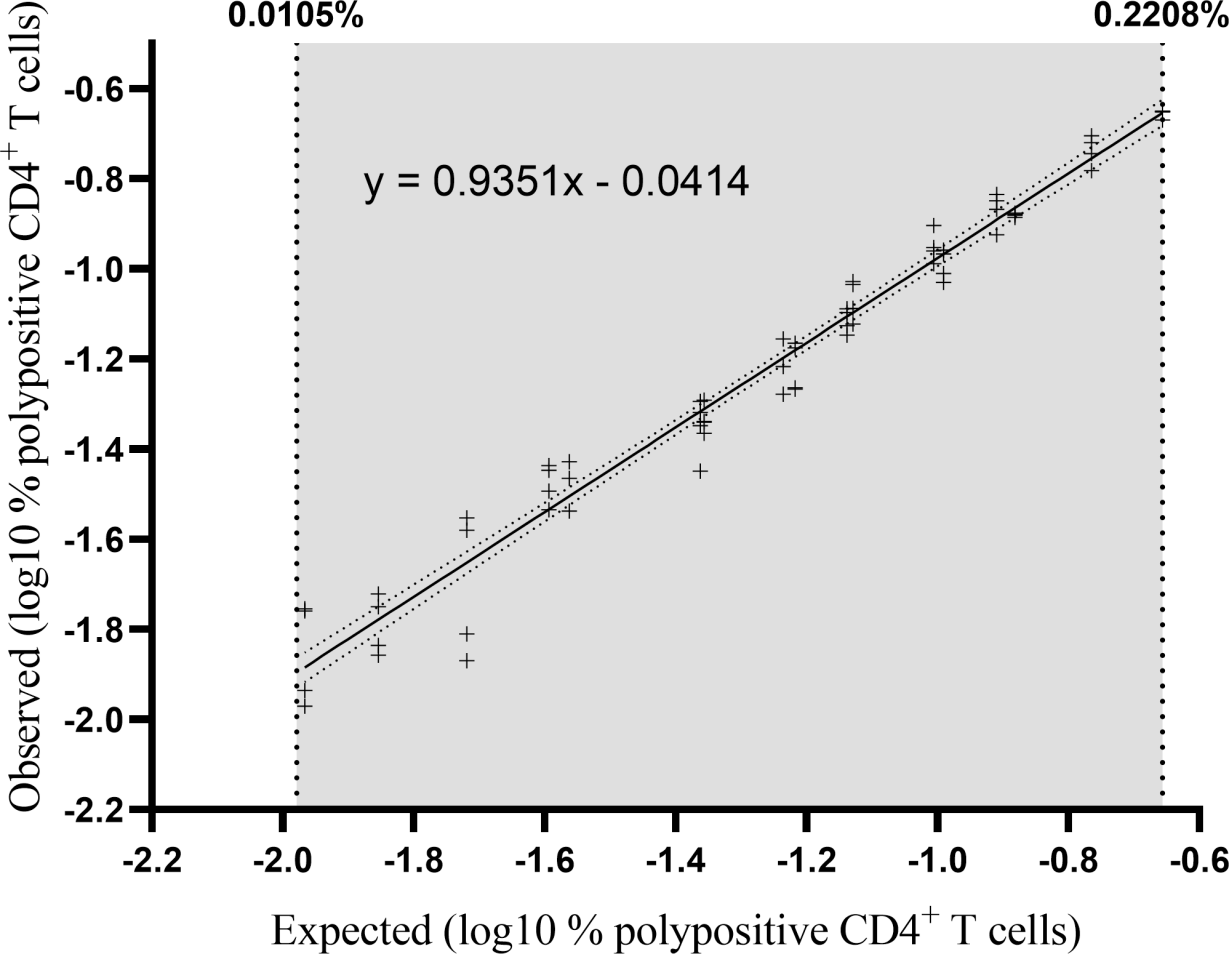
Percentage of observed versus expected corrected A/California/07/2009-specific polypositive CD4^+^ T cells. Individual datapoints represent pairs of background-corrected observed versus expected results of 68 individual results (3 samples [high, medium and low responders], 4 replicates, 5-6 independent dilutions). The results were log_10_ transformed and linearity was assessed by regressing the observed corrected against the expected corrected mean. The lower and upper limits of linearity are indicated with vertical dotted lines. The solid line represents the regression line and the dotted line represents the 95% confidence interval.

Three separate PBMC samples were diluted to five or six different concentrations (independent dilutions) and tested in quadruplicate in one single assay run (**Figure 6**). Linearity was demonstrated with a slope of 0.935 with a 90% CI of 0.90 to 0.97 and therefore included in the equivalence interval of 0.80 to 1.25 (**Table 3**). Relative accuracy was demonstrated, the mean recovery percentage was 111% with a 90% CI of 108% to 114%, and so included in the equivalence interval of 50% to 200%. The linearity and accuracy domain were between 0.0105% and 0.2208% polypositive CD4^+^ T cells (**Table 3**). The LLOL was 0.0105% polypositive CD4^+^ T cells (**Table 3**).

#### 3.3.4 Lower limit of quantification

LLOQ was the highest value between the LLOL and the LLOIP, and was set at 0.0335% polypositive CD4^+^ T cells, which was the LLOIP, and was determined in background-subtracted samples (**Table 3**).

#### 3.3.5 Specificity

**Figure 7:**
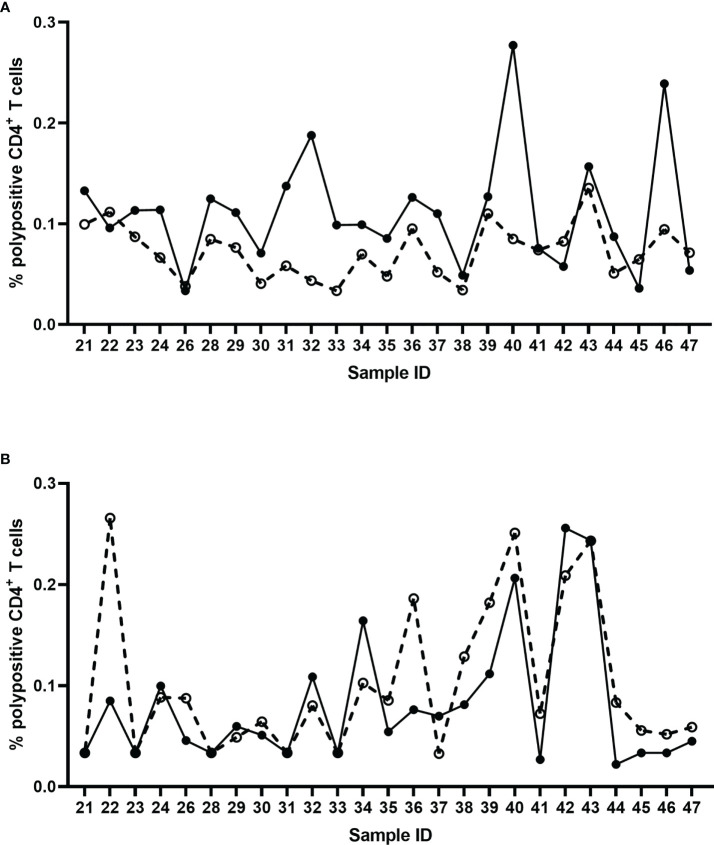
Percentage of corrected A/California/07/2009-specific **(A)** and non-specific (ATT) **(B)** polypositive CD4^+^ T cells before (dashed line) and 7 days after (solid line) seasonal influenza vaccination in 25 individual donors. Each data point represents an individual background-corrected result either pre- (open dots) or post- (closed dots) vaccination, reported as a percent of polypositive CD4^+^ T cells.

The specificity of the assay was tested using pre- and post-vaccination samples from 25 individual subjects by stimulating the PBMCs *in vitro* with an influenza vaccine antigen, monovalent influenza strain A/California/07/2009, or irrelevant, non-vaccine antigen (ATT). Specificity was demonstrated with a significant difference between pre- and post-vaccination result with the A/California stimulation (Wilcoxon signed-rank test, p<0.0001) (**Figure 7A**), and no significant difference between pre- and post-vaccination results with irrelevant antigen (Wilcoxon signed-rank test, p=0.0637) (**Figure 7B**; **Table 3**).

A summary of these qualification assay results is shown in **Table 3**.

## 4 Discussion

Many studies characterizing the cellular responses to influenza vaccines have used ICS alone or in combination with other assays (14–17). Quantifying and characterizing these cellular responses to vaccination is important for understanding longer term protection afforded by the vaccines. In this study we demonstrated the high variability between in-house ICS assays used by the seven participating laboratories, highlighting the concerns when comparing results from different studies obtained by different laboratories. Using the information collected during the pilot studies on the variety of parameters used for both the analytical and post-analytical stages of ICS, we were able to agree on critical parameters which should be fixed, and other, less critical parameters, where we leave flexibility to the laboratories. From this we were able to develop a consensus protocol for an optimized, and qualitatively and quantitatively qualified 8-color ICS assay. In this study, the selected antibody panel was chosen to detect a T helper 1 immune response and additional markers need to be added to detect other subpopulations such as T follicular helper or T regulatory cells. We have included a copy of this SOP as **Appendix 2**. The methods in this protocol have a great similarity to the flow cytometry guidelines proposed by Cossarizza et al. (18), and are furthermore similar to those reported in other recommendation for harmonization of the ICS method (8, 10). What we have shown here is the qualification of this harmonized protocol, providing detailed performance parameters.

This harmonized ICS assay has a very low limit of quantification and high sensitivity, is reproducible and linear, making it suitable for analysis of CMI responses in clinical trials of candidate influenza vaccines. The quantitative and qualitative data generated with this assay should enable more in-depth characterization of vaccine-induced T cell responses and could aid in determining if cellular immunity contributes to vaccine efficacy by potentially allowing the aggregation of data from different laboratories or trials.

This study allowed us to identify the parameters that vary between several in-house procedures and may have a critical impact on the outcome of the ICS assays. These variables that all lead to variation in responses are: thawing temperature, cell counting method, method applied to determine cell viability, concentration of cells per well, duration of cell incubation, and antibody panel. In this study we did not evaluate the impact of the stimulation medium, in particular any differences between serum-free and serum-containing media, or the impact of different serum batches. Both serum-free and serum-containing media were used in pilot study 1 by the participating laboratories, but this choice was not recorded; the qualification experiments were performed using serum-free AIM-V medium. Since this was not thoroughly investigated, the SOP recommends media containing validated batches of serum. Indeed, this could be one of the parameters that can help in further harmonizing the ICS assay. In our harmonized assay we chose to stain CD3 intracellularly. This approach was chosen because CD3 expression on the cell membrane is downregulated/internalized during stimulation and therefore, if stained after permeabilization, the CD3 molecules that are internalized are also stained. Additionally, the analysis of the acquired flow cytometry data can have a major impact on the accuracy of the final results. Indeed, most laboratories rely on the expertise of their FACS expert(s) to correctly set the “gates” and compensation values after having acquired the data. By identifying the imprecision introduced by data acquisition, and the inter-laboratory differences in the gating strategy (and compensation settings) we were able to develop guidelines to harmonize these procedures as well.

While we have developed a consensus protocol for ICS in the context of a response to an influenza vaccine, several limitations to the harmonization of ICS assays still remain. Different laboratories use different flow cytometers, with different configurations; so while the antibody clones can be standardized, the conjugated fluorochromes cannot be harmonized unless the read-out instruments are harmonized. Since different antigens can be used to elicit a specific immune response *in vitro* (such as living or inactivated viruses, virus like particles, recombinant antigens or peptides), experimental parameters may need to be adjusted and by consequence harmonized, accordingly. The lack of common inter-lab positive controls to normalize data across laboratories can also cause variation. Additionally, hands-on training for the harmonized protocol is important to maintain consistency. Hands-on training for this harmonized and qualified protocol was organized, and proficiency testing was performed subsequently. The outcome of this proficiency test will be presented in a separate paper.

In conclusion, we describe a harmonized and qualified ICS assay, which permits quantitative evaluation of CD4^+^ T cell responses induced by influenza vaccines. Application of this harmonized assay may allow future comparisons of T cell responses to different vaccines against influenza and other respiratory viruses and may ideally facilitate future assessments of potential correlates of protection.

## Data availability statement

The original contributions presented in the study are included in the article/**Supplementary Material**. Further inquiries can be directed to the corresponding author.

## Ethics statement

Ethical approvals for this study and the use of blood collected from Red Cross donors were given by the Ethical Committee of the Ghent University Hospital.

## Author contributions

SB, FC, GW, BS, GL-R, and AP contributed to conception and design of the study. SB, FC, GW, BS, MJ, DB, RJC, RD, EG, DM, EM, and EP contributed to the data acquisition, all authors contributed to the analysis and interpretation of the data. SB, FC, GW, and AP wrote the first draft of the manuscript. All authors contributed to critical revision of the manuscript, and read and approved the final version for submission.

## Funding

The FLUCOP project is supported by the Innovative Medicines Initiative Joint Undertaking (IMIJU) under grant agreement n° 115672, with financial contribution from the European Union Seventh Framework Programme (FP/2007-2013) and EFPIA companies’ in-kind contribution.

## Acknowledgments

The authors would like to acknowledge their FLUCOP consortium collaborators for their assistance; Marie-Clotilde Bernard, Caillet Catherine, Barbara Camilloni, Maria Rita Castrucci, Marco Cavaleri, Simon De Lusignan, Oliver Dibben, Othmar Engelhardt, Susanna Maria Roberta Esposito, Marzia Facchini, Felipa Ferreira, Sophie Germain, Sarah Gilbert, Sammy Ho, Katja Hoschler, Sarah L Jalloh, Stefan Jungbluth, Marion Koopmans, Teresa Lambe, Manuela Mura, Nedzad Music, Martina Ochs, Thierry Ollinger, Albert Osterhaus, Giuseppe Palladino, Remarque Ed, Leslie Reperant, Hanna Sediri-Schön, Alexandre Templier, Sarah Tete, Claudia Trombetta, Serge Van de Witte, Ralf Wagner, Joanna Waldock, Brenda Westerhuis, and Fan Zhou. They would also like to thank Joseline Ruiz (Sanofi) for the statistical analysis of the qualification assay. Editorial assistance with the preparation of the manuscript was provided by Nicola Truss, PhD of inScience Communications, Springer Healthcare, London, UK. Funding for this assistance was provided by Sanofi. The authors also thank Isabel Grégoire for providing editorial assistance and manuscript coordination on behalf of Sanofi. The authors thank Teresa Lambe (University of Oxford) for her contribution to the experimental approach, strategy, analysis and delivery of the ICS results.

## Conflict of interest

Authors EM and EG are employees of VisMederi srl. MJ, and BS are employees of GlaxoSmithKline. SB and AP are employees of Sanofi and may hold stock.

The remaining authors declare that the research was conducted in the absence of any commercial or financial relationships that could be construed as a potential conflict of interest.

The authors declare that this study received funding from Sanofi. The funder had the following involvement in the study: analysis and interpretation of the data, the writing of this article, and the decision to submit for publication

## Publisher’s note

All claims expressed in this article are solely those of the authors and do not necessarily represent those of their affiliated organizations, or those of the publisher, the editors and the reviewers. Any product that may be evaluated in this article, or claim that may be made by its manufacturer, is not guaranteed or endorsed by the publisher.
