# Supplementary material for: Harmonization and qualification of intracellular cytokine staining to measure influenza-specific CD4+ T cell immunity within the FLUCOP consortium
Source: Front Immunol. 2022 Oct 20;13:982887. doi: 10.3389/fimmu.2022.982887 (PMC9632653; doi:10.3389/fimmu.2022.982887)

#
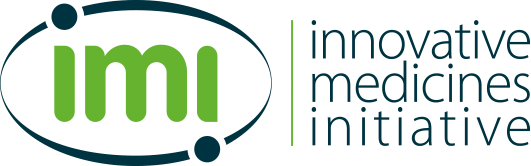


**
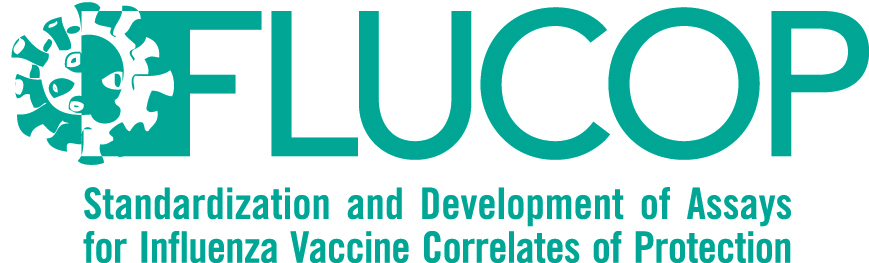
**

**Standard Operating Procedure for Intracellular Cytokine Staining (ICS)**

Table of Contents

[1](#_Toc48288132)

[INTRODUCTION 3](#_Toc48288133)

[REAGENTS 4](#_Toc48288134)

[MATERIALS and EQUIPMENT 4](#_Toc48288135)

[TRAINING AND PROFICIENCY 4](#_Toc48288136)

[PROCEDURES 5](#_Toc48288137)

[Thawing of cells and stimulation, Day 1 5](#_Toc48288138)

[Fixation, permeabilisation and staining. Day 2 6](#_Toc48288139)

[Acquisition of data 8](#_Toc48288140)

[Data analysis 8](#_Toc48288141)

# INTRODUCTION

The overall objective of Work Package (WP) 2 within the FLUCOP project was to advance the understanding and application of cell-mediated immunity (CMI) assays as tools for evaluating the immunogenicity of influenza vaccines. One of the tasks was to create a standardized protocol on how to perform the Intracellular Cytokine Staining (ICS) assay.

Cytokine production plays an important role in the immune response. Cytokines are involved in many different pathways including the induction of many anti-viral proteins by interferon-gamma (IFN-γ), the induction of T cell proliferation by IL-2 and the inhibition of viral gene expression and replication by tumour necrosis factor-alpha (TNF-α). Cytokines are not preformed factors but are rapidly produced and secreted in response to cellular activation.

ICS is a widely used flow cytometry-based assay that detects the production and accumulation of cytokines within the Golgi apparatus and/or endoplasmic reticulum after cell stimulation. ICS can be used in combination with other flow cytometry protocols for immunophenotyping using cell surface markers or with MHC multimers to detect an antigen-specific response, making it an extremely flexible and versatile method.

The principle of intracellular cytokine staining is as follows:

- Cells are activated using either a specific or non-specific activation cocktail
- An inhibitor of protein transport (e.g. brefeldin A) is added to retain the cytokines within the cell
- After washing, antibodies to other cellular markers can be added to the cells
- The cells are then fixed in paraformaldehyde and permeabilized
- The anti-cytokine antibody is added and the cells can be analysed by a flow cytometer

The enumeration of polypositive CD4^+^ T cells has been selected based on results obtained in the FLUCOP WP2 project as being the preferred read-out allowing the quantification of present cell-mediated immunity in samples obtained after natural infection or vaccination. Polypositive meaning any CD3^+^/CD4^+^ cells or CD3+/CD8+ cells positive for at the least two of the following parameters: IFN-γ, TNF-α, IL-2 and CD154.

# REAGENTS

Reagents are critical in the ICS assay. It is important to work with high-quality peripheral blood mononuclear cells (PBMC) samples. The tissue culture media (complete RPMI or cRPMI) need to contain sufficient nutrients to safeguard the structural and functional integrity of the cells and allow them to respond adequately upon activation. Foetal bovine serum should be validated before use to check whether it provides sufficient support without generating undesirable background noise.

It is good practice to trace the lot numbers of the used reagents, their expiration dates and certificates of analysis.

# MATERIALS and EQUIPMENT

Using good quality lab materials will ensure a good quality of the processed sample. Plastic disposables need to be of lab-grade quality and sterile (packed individually). Changing suppliers or brands should trigger a change control process to avoid accidental misfortune and subsequent sample loss. Equipment is crucial for the success of the process. These should be well designed and fully qualified (based on the method suitability and established standard) before taking into production. Proper control maintenance and requalification after technical intervention should assure proper performance throughout their lifecycle.

# TRAINING AND PROFICIENCY

It is good practice first to train the technician and obtain proof of their proficiency before engaging the person in conducting ICS assays for clinical trials purposes.

# PROCEDURES

| General recommendations:  FLUCOP recommends to execute the Intracellular Cytokine Staining assay for monitoring of cellular immune responses in the frame influenza vaccine trails under the control of a proper Quality Assurance program.  Available guidelines are :   - ICH E6 R2 Guidance for Industry, Good Clinical Practice - Good Clinical Laboratory Practice (GCLP) Version 2, BARQA, Tim Stiles Vanessa, Grant Nick Mawby , ISBN 978-­‐1-­‐904610-­‐00-­‐7 - EMA/INS/GCP/532137/2010 28 February 2012 Reflection paper for laboratories that perform the analysis or evaluation of clinical trial samples |
| --- |

## Thawing of cells and stimulation, Day 1

- Prepare one 15 mL conical tube per sample that will be analyzed in the laminar flow bench: label and fill with 7 mL cRPMI.
- Immediately transfer the cryovials from a liquid nitrogen-filled container to the water bath (37°C ± 1°C). Thaw the cryovials in small batches at a time.
- Transfer the PBMC to a 15 mL conical tube filled with 7 mL cRPMI.
- Centrifuge at 350 g for 7 minutes at room temperature (RT).
- Remove the supernatant from the 15 mL conical tubes.
- Resuspend the cells in 5 mL cRPMI benzonase solution (cRPMI with benzonase at a concentration of 25 units/mL).
- Incubate for 10 minutes in a water bath (37°C ± 1°C).
- Centrifuge at 350 g for 7 minutes at RT.
- Remove the supernatant.
- Resuspend the cells in 2 mL cRPMI and count the cells and determine cell viability.

| Recommendation:  Cell counting can be executed using manual or automated methods. However, it is important to validate the selected method, and to evaluate the proficiency of the executing operators. |
| --- |

- Centrifuge the cells at 350 g for 7 minutes at RT.
- Remove the supernatant from the washed cells.
- Resuspend the cells in cRPMI at a selected final cell concentration.

Note: take into account the volume remaining with the cell pellet when resuspending the cells

| Recommendation:  FLUCOP recommends stimulating cells at a concentration 20 x10^6^/mL, meaning 1 million cells per well. |
| --- |

- Transfer cells and stimulants to the U-bottom plates as follows:
- For each sample, add 50 μL of antigen/peptide to the corresponding well. The antigen/peptide should be at optimal concentration (taking into account the dilution factor).
- For each sample prepare at least 2 wells: one background or unstimulated well and one well per stimulation condition. Note, in case of stimulation with the superantigen SEB, physically separate the SEB-stimulated wells from regular wells to avoid cross-contamination.

| Recommendations:  Stimulating agents (peptides, proteins, virus-like particles (VLPs),…) should be pre-tested to evaluate the optimal stimulation concentration and potential cell cytotoxicity. |
| --- |

- Prepare the co-stimulating CD28-CD49d solution in cRPMI at a final concentration of 1 μg/mL (e.g. at a dilution of 1/250). Add 50 μL to **all** wells.
- Add 50 μL of the cell suspension to the corresponding wells.
- Incubate for 2 hours at 37°C (± 1°C) and 5% CO_2_ in a humid atmosphere.
- Prepare the protein transport inhibitor, Brefeldin A, solution. Add 50 μL to **all** wells
- Cover the plates with porous micropore tape and incubate for approximately 18 hours at 37°C (± 1°C) and 5% CO_2_ in a humid atmosphere.

## Fixation, permeabilisation and staining. Day 2

- Prepare DPBS+1%FBS with filtered FBS.
- If applicable: thaw the DMSO vial and a vial of the fixable Live/Dead dye. Keep the Live/Dead dye vial protected from light.
- Calculate the quantities required (50 μL/sample/well + 1 well for an all-colors well) for the extracellular antibody cocktail (e.g CD4/CD8/Live-Dead). The antibodies must be used at optimal concentration.
- Transfer the cells to a 96-well V-bottom plate.
- Centrifuge for 5 minutes at 350 g.
- If applicable: Prepare the LIVE/DEAD stock solution by adding 50 μL DMSO to the LIVE/DEAD vial.
- Remove supernatant from the plates. Where applicable, treat the SEB plate last.

Note: When using a manifold aspirator, rinse it with lab grade water or change tips to avoid contamination with SEB

- Resuspend the cells using 50 μL extracellular antibody cocktail (e.g. CD4-CD8-LIVE/DEAD).

| Recommendation :  FLUCOP recommends to perform the CD3 staining in the intracellular staining step. This results in a brighter CD3 staining as this enables staining of the internalized CD3 molecules. |
| --- |

- Incubate for 20 minutes (± 4 minutes) at RT in the dark.
- Add 125 μL DPBS+1%FCS to all wells.
- Centrifuge for 5 minutes at 350 g at RT.
- Remove the supernatant.
- Wash the cells with 175 μL DPBS+1%FCS.
- Centrifuge for 5 minutes at 350 g at RT.
- Remove the supernatant.
- Resuspend all cells using 175 μL Cytofix/Cytoperm
- Incubate for 20 minutes (± 1 min) at RT, in the dark.
- Prepare Perm/Wash solution by diluting 1:10 in lab-grade water
- Centrifuge for 5 minutes at 500 g at RT.
- Remove the supernatant.
- Wash the cells with 175 μL Perm/Wash.
- Centrifuge for 5 minutes at 500 g at RT.
- Prepare the necessary volumes (50 μL/sample/well + 1 well for all-color well) of the intracellular antibody cocktail.

This solution will contain following conjugated antibodies diluted in Perm/Wash:

- Anti-human CD3 antibody
- Anti-human IFN-γ antibody
- Anti-human IL-2 antibody
- Anti-human CD154 antibody
- Anti-human TNF-α antibody

| Recommendation:  FLUCOP recommends the following antibody clones for the above-mentioned antibody panel:   - - Anti-human CD3 antibody – UCHT1 - Anti-human CD4 antibody – SK3 - Anti-human CD8 antibody – SK1 - Anti-human IFN-γ antibody – 4S.B3 - Anti-human IL-2 antibody – MQ1 17H12 - Anti-human CD154 antibody – TRAP-1 - Anti-human TNF-α antibody – Mab11   The choice of fluorochrome should be based on proper flow cytometry principles. Herein the spectral overlap, antigen density and brightness of the fluorochrome should be taken in account. |
| --- |

- Remove the supernatant.
- Resuspend the cells using 50 μL of extracellular antibody cocktail (e.g. CD3, IL-2, IFN-γ, CD154, TNF-α)
- Incubate for 20 minutes at RT in the dark.
- Add 125 μL Perm/Wash to all wells.
- Centrifuge for 5 minutes at 500 g at RT.
- Remove the supernatant.
- Resuspend the cells using 230 μL DPBS+1%FCS.
- The plates can be kept at 2-8°C in the dark until read-out takes place.

## Acquisition of data

- Acquire data on a flow cytometer equipped with sufficient lasers and detectors to enable acquisition without extreme spectral overflow of signals. Make sure to include proper compensation routines in every experiment and at change of any assay- or instrument parameters.
- Acquisition threshold can be set to avoid acquiring ‘debris’ data
- Collect all cellular data

Recommendations:

- use the Forward Scatter to further discard all non-cellular debris.
- Acquire at the least 75 000 Live CD3^+^CD4^+^ parent cells. Optimally 100 000 CD4^+^ T cells should be acquired.

## Data analysis

| Recommendations:   - Analyse stability of the acquisition process by analysing a dot-plot showing time vs one of the following parameters: IFN-γ, TNF-α, IL2 or CD154 - Select Single events by gating in a FCS area vs FCS wide dot plot - Where needed use density or ‘zebra’ plots to enable proper gating - Show proper care not to ‘over use’ bi-exponential gating - Set gates as such to avoid losing positivity by CD3 internalisation - Apply back-gating on high positive cells to validate all sublevel gatings - Use well-defined flagging codes to indicate if an error occurred or if certain predefined quality criteria have not been met. |
| --- |

Analyse the data and enumerate the number of CD3^+^CD4^+^ T cells positive for at the least 2 of the following parameters: CD154, IFN-γ, TNF-α, IL-2. Polypositivity can be determined via Boolean combinations:


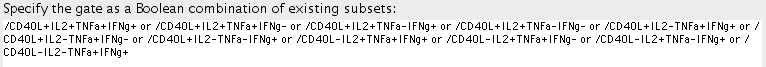


# **APPENDIX 1:** Gating strategy

Example of the gating strategy for Flu vaccine H1N1 A/California/07/2009 strain (Appendix 2) or Staphylococcal enterotoxin B (SEB) (Appendix 3) stimulated PBMCs.

(A): Initial gating is done on FSC-A and Time (Time gate). In this example, events collected at the very beginning of acquisition when fluctuations may occur were excluded. In the template, we chose to have 2 Time gates to keep the possibility to exclude cells at later acquisition times in case fluctuations occur. The two “Time gates” are combined using a mathematical “or” association and on this population, single cells are selected using FSC-H and FSC-A gate. Dead cells are excluded using an amine reactive dye (Fixable Aqua LIVE/DEAD AViD). The lymphocytes are loosely gated using a drawn gate in an FSC-A (size) and SSC-A (granularity) plot. CD3+ cells are gated using a CD3 BV786 vs. CD154 (CD40L) PE plot to make sure all CD154+, including those showing a CD3^dim^ expression, are selected. Within this CD3+ lymphocyte gate, CD4 and CD8 T cells are identified.

(B): Functional markers for CD4 and CD8 T cells. A gate is applied for each cytokine, not taking into account the co-expression of other markers (SSC-A versus Cytokines/Marker). Then Boolean gates are created based on these gates to identify cells expressing different combinations of markers. CD154 was excluded from the Boolean gate’s calculation within the CD8 T cell population. The CD4 polypositive T cells and CD8 polypositive T cells are the cells secreting at least 2 cytokines/activation marker.

For a same donor, all the gates for functional markers were defined using the unstimulated sample (Medium). For each file and each donor, different processes are done to ensure having the best noise/antigen specific response as:

1. The position of the quadrants on Cytokine X versus Cytokine Y are used to verify the position of each cytokine/marker gate (SSC-A versus XX) for each file – see examples in (C) for the IFN-γ secreted CD4 and CD8 cells.
2. for each donor using the SEB (POSITIVE CONTROL) as well.
3. A backgating process done on each “cytokine positive gate” ensure that all cytokine secreted cells (as dim cells) are included in the gates drawn along the strategy analysis.

(D): All the gating strategy used on the FCS qualification files begins with a Time gate design on FSC-A/Time dot plot to identify and remove periods of time during the run where bubbles, clogs, or dry air were introduced. For the proficiency ICS test, it was recommended to have or to verify the acquisition on a representation of the Time gate drawn on a dot plot “Time versus a cytokine” that allowed the see on a fluorescent marker when a clog or bubbles can influence the light and introduce what looks like false positive quantification. Usually, we verify this on each laser using the last channel used in flow cytometer.

# **APPENDIX 2**: Example of gating strategy of H1N1 A/California/07/2009 stimulated PBMC sample


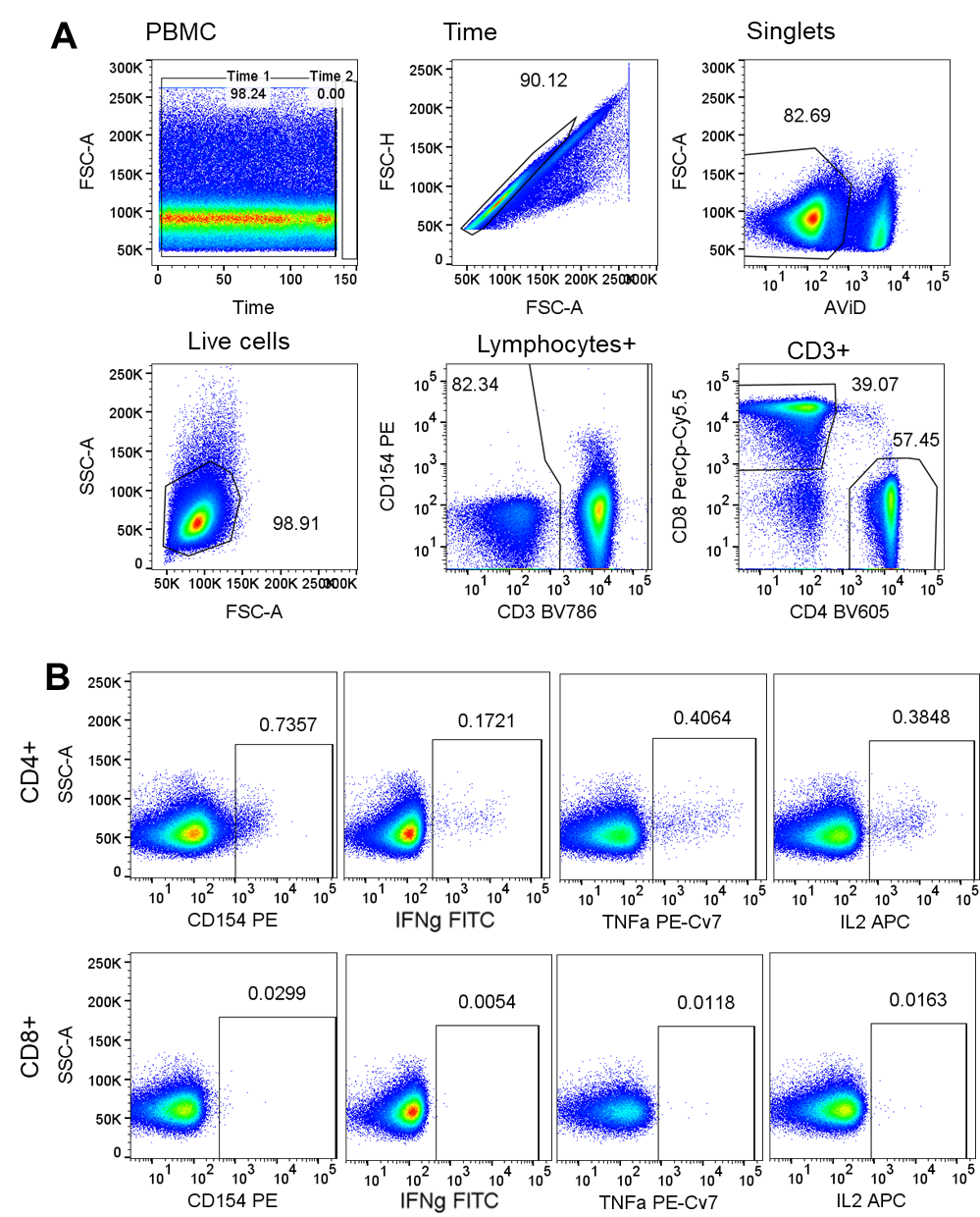


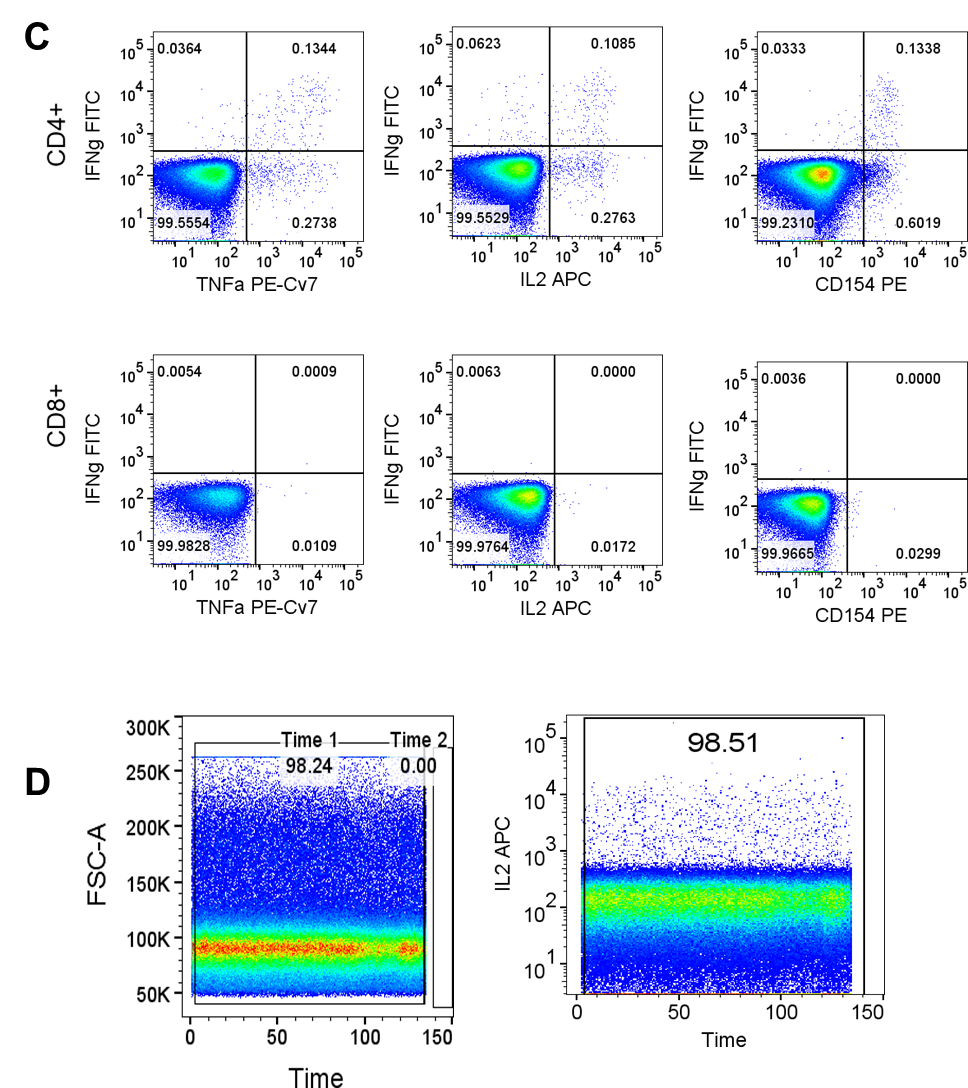


# **APPENDIX 3:** Example of gating strategy of Staphylococcus Enteroxin B (SEB) stimulated PBMC sample

#
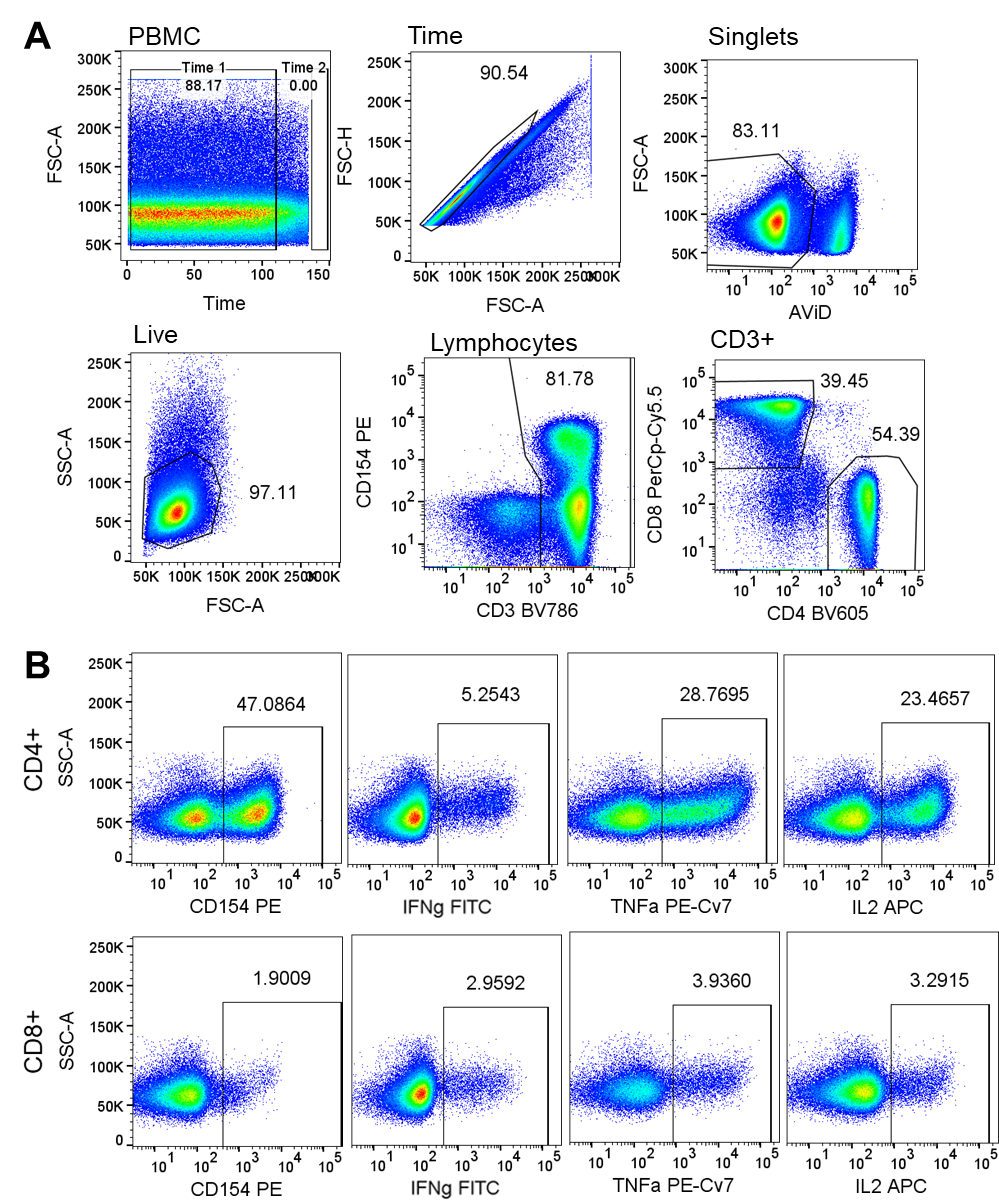


CD4+

**C**

CD8+


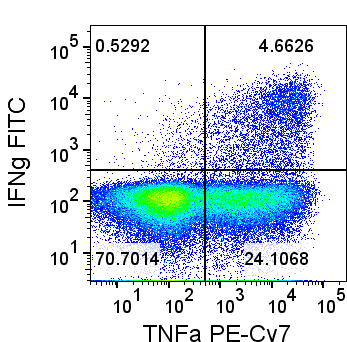

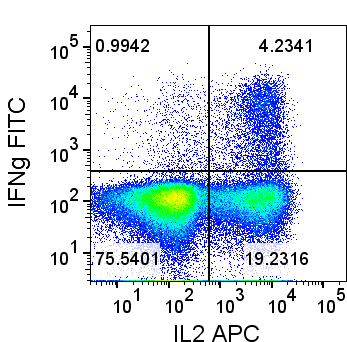

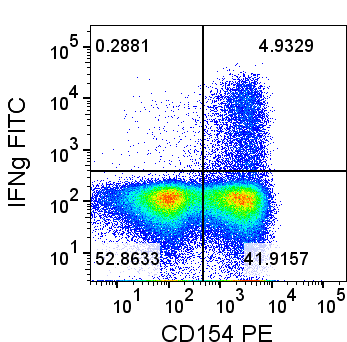

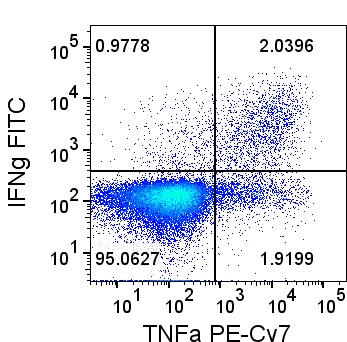

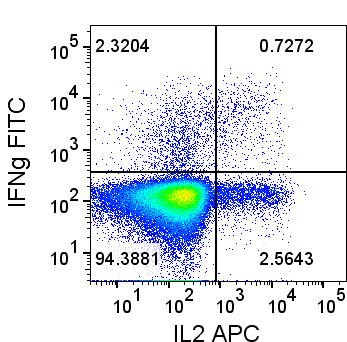

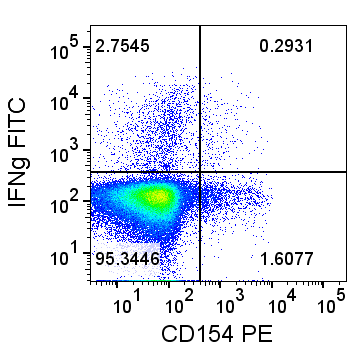

Supplement: Supplementary file 2 [file DataSheet_2.docx]
