# Supplementary material for: Harmonization and qualification of intracellular cytokine staining to measure influenza-specific CD4+ T cell immunity within the FLUCOP consortium
Source: Front Immunol. 2022 Oct 20;13:982887. doi: 10.3389/fimmu.2022.982887 (PMC9632653; doi:10.3389/fimmu.2022.982887)
Supplement: Supplementary file 3 [file DataSheet_3.docx]

***Supplementary Materials***

**FLUCOP Consortium Collaborators**

Sanofi

Joseline Ruiz: statistical analysis for qualification of method

University of Ghent

Pilot study 1: Sabrina Verlee, Sanne Foubert

Pilot study 2: Peter Vander Linden

University of Bergen

Rebecca Jane Cox, Richard Davies

VisMederi

Alessandro Torelli, Ilaria Razzano

UNISI

Annalisa Ciabattini

**Supplementary Table 1.** Parameters reported by each laboratory in pilot study 1

| **Parameters** |
| --- |
| Reported in an Excel file:  Name of the laboratory  Stimulating agent  Mortality [percentage of dead cells]  Sample identification  Marker  Events/parent events [frequency]  Counted parent events |
| Reported in an online portal:  Number of vials thawed  Thawing medium  Thawing medium temperature  Thawing  Fetal bovine serum (FBS) validation status  Cell counting techniques  Applied technique for determination of cell viability  Cell concentration in the well  Resting time  Stimulation/incubation time  Incubation conditions  Protein transport inhibitor used  Lyse/fix reagent  Permeabilizing reagent  Duration of antibody staining  Intracellular (IC) staining  Extracellular (EC) staining  Acquisition  Flow cytometer  Quality control (QC) processes for the flow cytometer  Photomultiplier tubes (PMT) voltages  Compensation  Threshold during acquisition  Validation criteria on background conditions  Any comments/deviations |

**Supplementary Table 2.** Staining in the provided flow cytometry data for pilot study 2

| **Parameter** | **Antibody Clones** | **Fluorochrome** |
| --- | --- | --- |
| Forward Scatter | NA | NA |
| Side Scatter | NA | NA |
| Live/Dead stain | NA | Aqua |
| CD3 | UCHT1 | AlexaFluor700 |
| CD4 | SK3 | PerCPCy5.5 |
| CD8 | SK1 | APC-H7 |
| IFNγ | 4S.B3 | FITC |
| IL2 | MQ1-17H12 | APC |
| TNFα | Mab11 | PE-Cy7 |

APC, allophycocyanin; Cy, cyanine; FITC, fluorescein isothiocyanate; IFN, interferon; IL, interleukin; NA, not applicable; TNF, tumor necrosis factor; PE, R-phycoerythrin; PerCP, peridinin-chlorophyll-protein complex.

**Supplementary Table 3.** Flow cytometry staining for the qualification of the harmonized SOP

| **Staining step** | **Specificty** | **Clone** | **Fluorochrome** | **Manufacturer (reference)** |
| --- | --- | --- | --- | --- |
| Surface | Dead cells | NA | Live/Dead Fixable Aqua | MolecularProbes  (L34957) |
| Surface | Anti-human CD4 | SK3 | BV605 | BioLegend (344646) |
| Surface | Anti-human CD8 | SK1 | PerCpCy5.5 | BioLegend (344710) |
| Intracellular | Anti-human CD3 | UCHT1 | BV786 | Becton Dickinson (565491) |
| Intracellular | Anti-human IFNγ | 4S.B3 | FITC | BioLegend (502506) |
| Intracellular | Anti-human IL-2 | MQ1-17H12 | APC | BioLegend (500310) |
| Intracellular | Anti-human TNFα | Mab11 | PE-Cy7 | BioLegend (502930) |
| Intracellular | Anti-human CD40L (CD154) | TRAP1 | PE | Becton Dickinson (555700) |

APC, allophycocyanin; Cy, cyanine; FITC, fluorescein isothiocyanate; IFN, interferon; IL, interleukin; TNF, tumor necrosis factor; PE, R-phycoerythrin; PerCP, peridinin-chlorophyll-protein complex.

**Supplementary Table 4.** Instrument configuration used for the ICS qualification

| **Laser wavelength [nm]** | **Laser power [mW]** | **Laser type** | **Spectral range for detector [nm]** | **Dichroic mirror [nm]** | **Band pass [nm]** | **Fluorochrome**** |
| --- | --- | --- | --- | --- | --- | --- |
| 488 | 20 | DPSS* | 750-810 | 755 LP | 780/60 | PE-Cy7 |
|  |  |  | 675-715 | 685 LP | 695/40 | PerCP-Cy5-5 |
|  |  |  | 650-670 | 655 LP | 660/20 | PE-Cy5 |
|  |  |  | 600-630 | 600 LP | 610/20 | PE-CF594 |
|  |  |  | 562-588 | 550 LP | 575/26 | PE |
|  |  |  | 515-545 | 505 LP | 530/30 | FITC |
| 405 | 60 | DPSS* | 750-810 | 750 LP | 780/60 | BV786 |
|  |  |  | 685-735 | 690 LP | 710/50 | BV711 |
|  |  |  | 650-670 | 630 LP | 660/20 | BV650 |
|  |  |  | 600-620 | 595 LP | 610/20 | BV605 |
|  |  |  | 500-550 | 505 LP | 525/50 | AquaBlue |
|  |  |  | 425-475 | None | 450/50 | BV421 |
| 633 | 25 | DPSS* | 750-810 | 755 LP | 780/60 | APC-Cy7 |
|  |  |  | 707.5-752.5 | 710 LP | 730/45 | Alexa Fluor 700 |
|  |  |  | 650-670 | None | 660/20 | APC |

*** *DPSS, diode-pumped solid-state laser.*

*** APC, Allophycocyanin; BV, Brilliant Violet; PE, Phycoerythrin; Cy, cyanine; FITC, fluorescein isothiocyanate; PerCP, peridinin-chlorophyll-protein complex.*

The qualification panel was optimized using an LSRII cytometer (BD Biosciences) with a three-laser configuration and the listed optical elements. For each laser, the long pass (LP) dichroic filter in each detector sends longer wavelengths through the respective band pass filter and reflects shorter wavelengths down the optical path to the next dichroic filter.

**Supplementary Figure 1:** The gating strategy used to analyze the FCS files for qualification experiments, using the gating strategy for Flu vaccine H1N1 A/California/07/2009 strain or Staphylococcal enterotoxin B (SEB) stimulated PBMCs as an example

(A): Initial gating is done on FSC-A and Time (Time gate). In this example, only events collected at the very beginning of acquisition when fluctuations may occur were excluded. We chose to have 2 Time gates to keep the possibility to avoid fluctuation during a late acquisition time as well as we couldn’t apply such algorithm like FlowAI to clean our data at that time. The single cells are selected using FSC-H and FSC-A gate after a mathematical “or” association between Time 1 / Time 2 gates. Dead cells are excluded using an amine reactive dye (Fixable Aqua LIVE/DEAD aka AViD). The lymphocytes are gated using a drawn gate using FSC-A (size) and SSC-A (granularity). Subsequent gating discriminates the antigen activated T lymphocytes by CD3 BV786 and CD154 (CD40L) PE expression (CD3^+^ cells that also expressed CD154^+^ marker). Within this CD3^+^ lymphocyte gate, CD4 and CD8 T cells are identified.


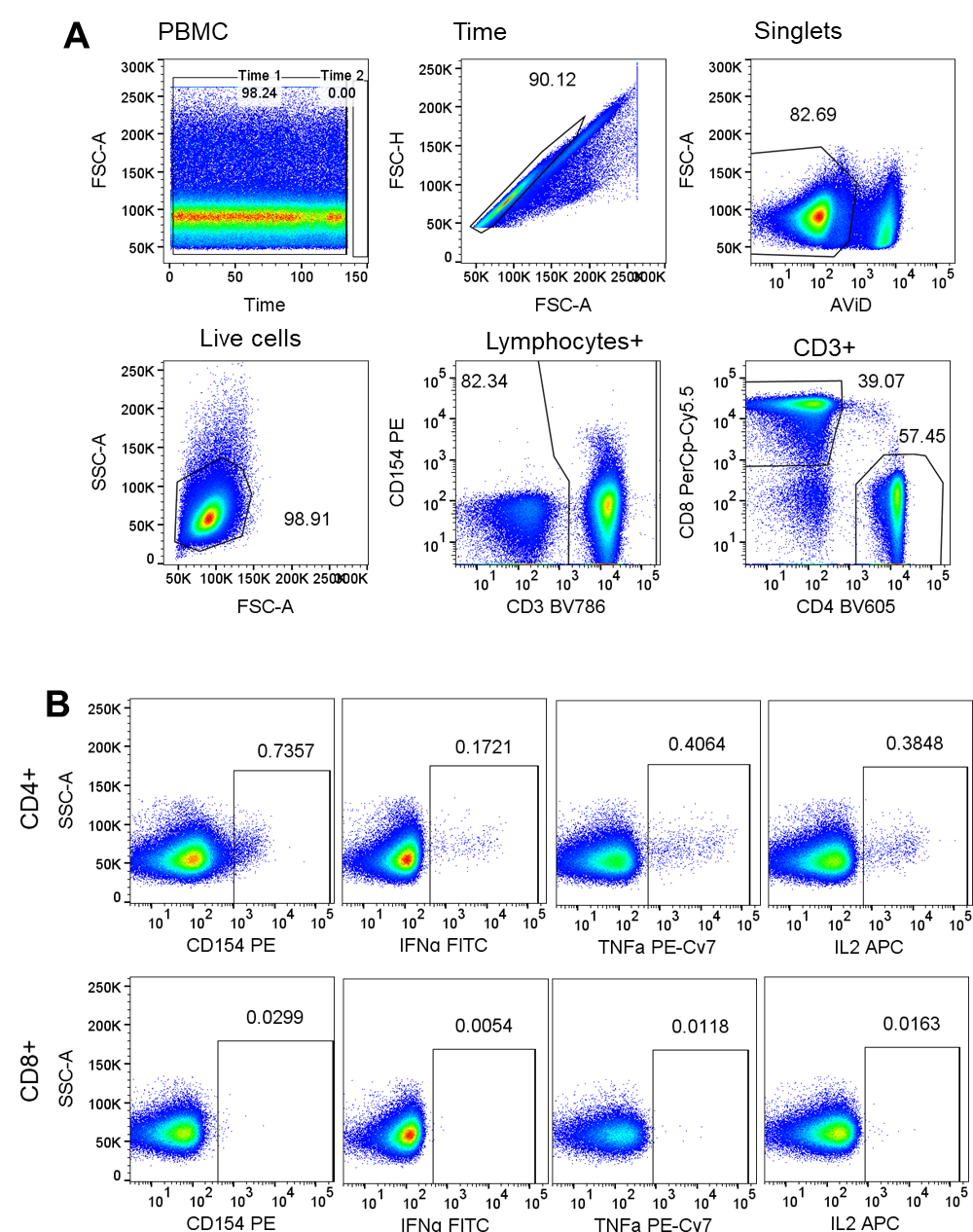


(B): Functional markers for CD4 and CD8 T cells. A gate is applied for each cytokine, not taking into account the co-expression of other markers (SSC-A versus Cytokines/Marker). Then Boolean gates are created based on these gates to identify cells expressing different combinations of markers. CD154 was excluded from the Boolean gate’s calculation within the CD8 T cells population. The CD4 polypositive T cells and CD8 polypositive T cells are the cells secreting at least 2 cytokines/activation marker. For a same donor, all the gates for functional markers were defined using the unstimulated sample (Medium). For each file and each donor, different processes are followed to ensure having the best signal/antigen specific response as:

1. The position of the quadrants on Cytokine X versus Cytokine Y are used to verify the position of each cytokine/marker gate (SSC-A versus XX) for each file – see examples in (C) for the IFN-γ secreted CD4 and CD8 T cells.
2. for each donor using the SEB (POSITIVE CONTROL) as well.
3. A backgating process on each “cytokine positive gate” ensuring that all cytokine secreted cells (such as dim cells) are included in the gates drawn along the strategy analysis.


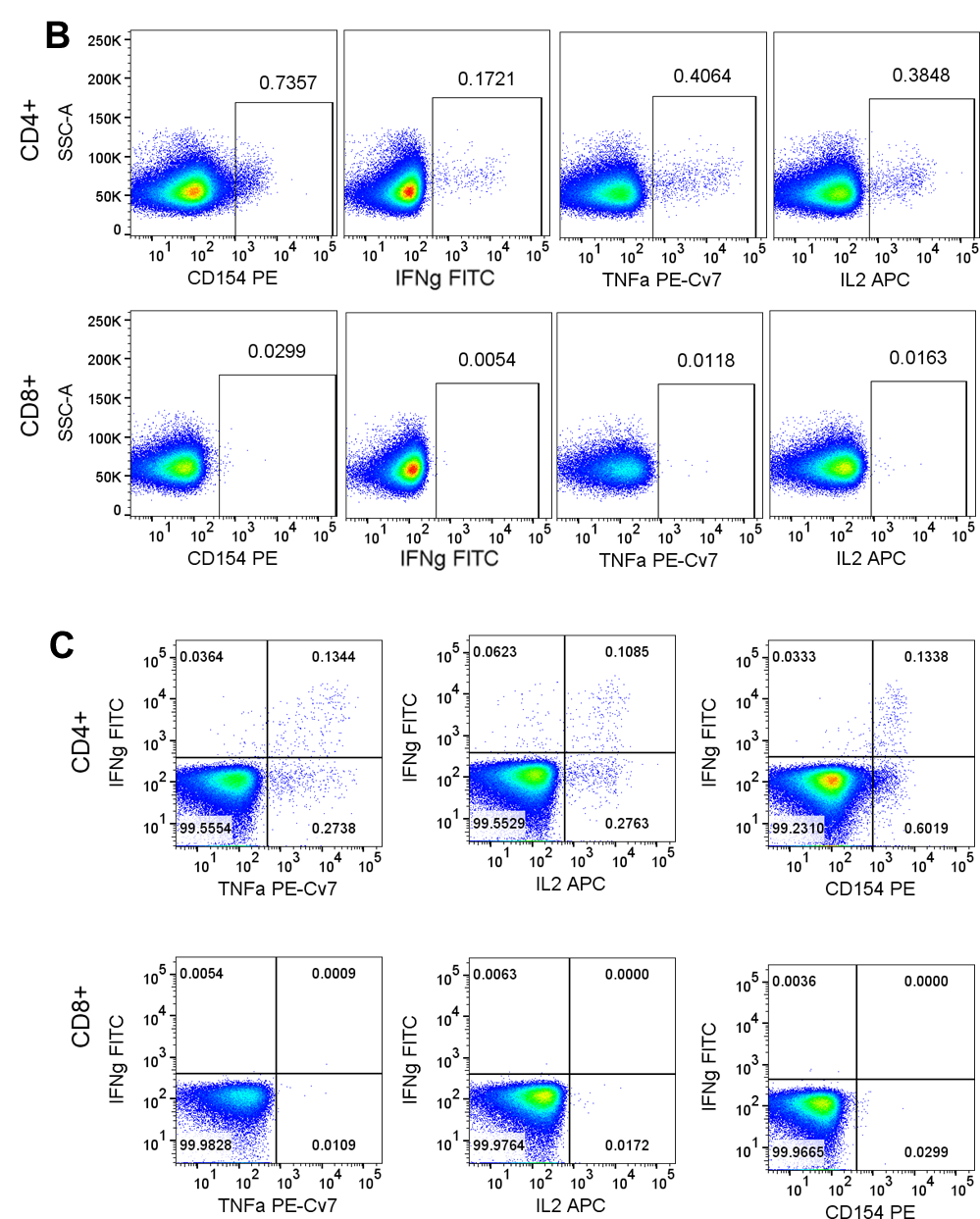


(D): All the gating strategy used on the FCS qualification files begins with a Time gate design on FSC-A/Time dot plot to identify and remove periods of time during the run where bubbles, clogs, or dry air were introduced. For the proficiency ICS test, it was recommended to have or to verify the acquisition on a representation of the Time gate drawn on a dot plot “Time versus a cytokine” that allowed the see on a fluorescent marker when a clog or bubbles can influence the light and introduce what looks like false positive quantification. Usually, we verify this on each laser using the last channel used in flow cytometer.


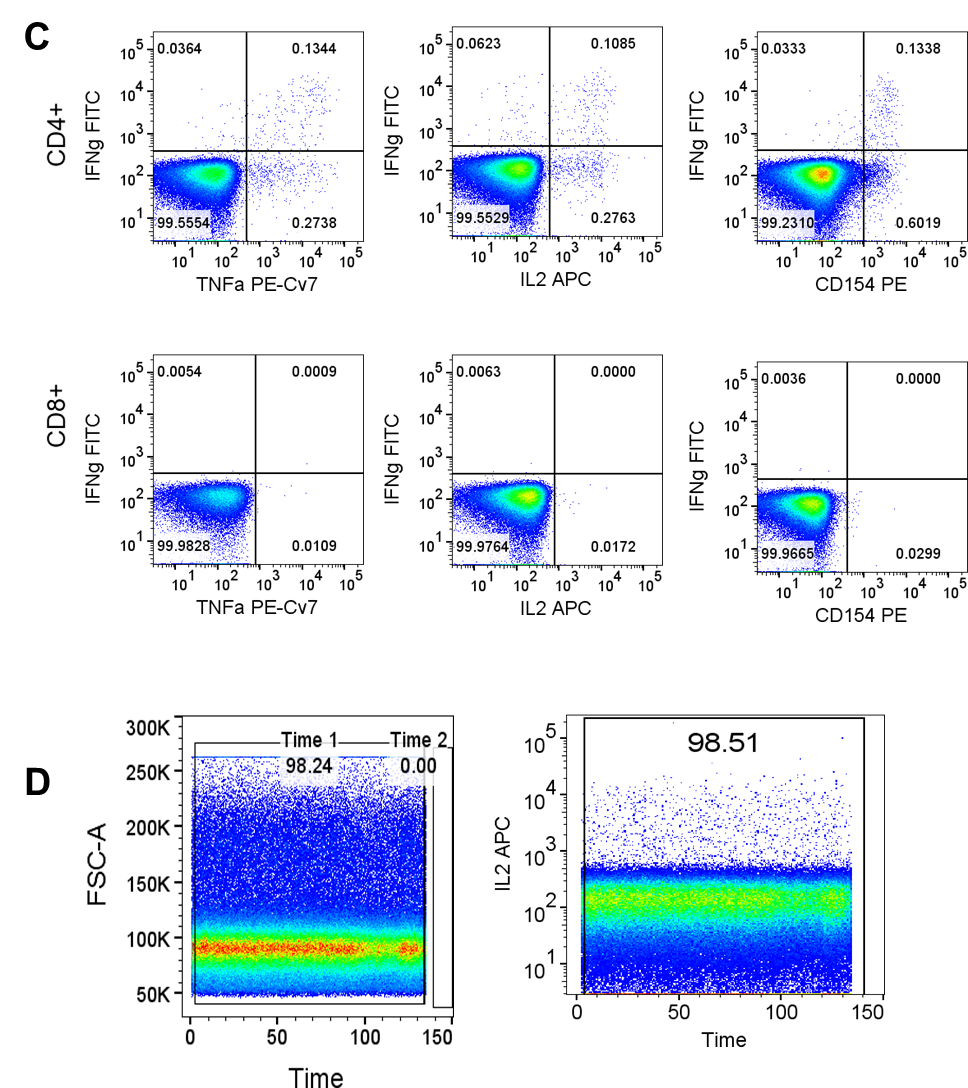


**Supplementary Figure 2:** Gating Strategy applied by the 8 laboratories in Pilot Study 2

| **1** | **2** | **3** | **4** | **5** | **6** | **7** | **8** |
| --- | --- | --- | --- | --- | --- | --- | --- |
| 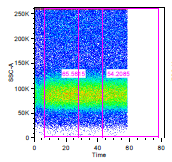 | 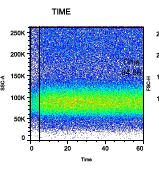 | 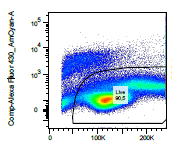 | 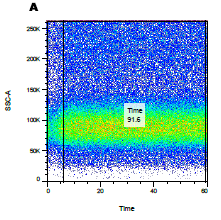 | 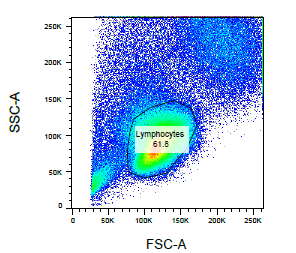 |  | 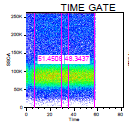 | 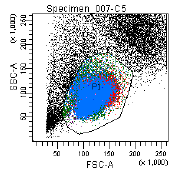 |
| 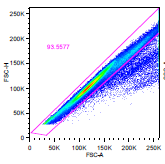 | 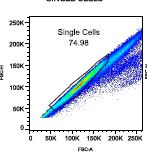 | 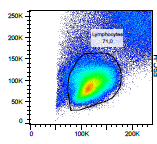 | 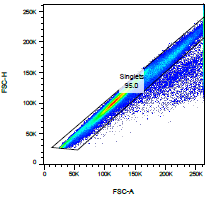 | 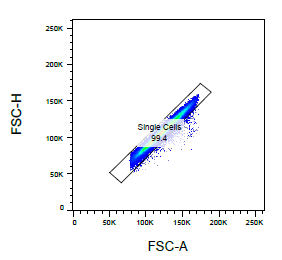 |  | 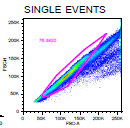 | 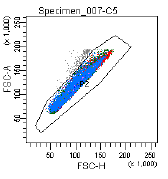 |
| 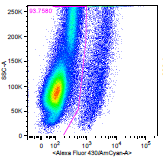 | 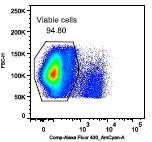 | 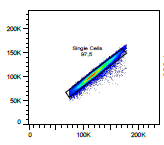 | 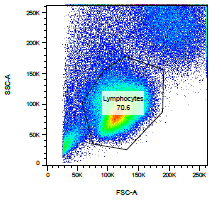 | 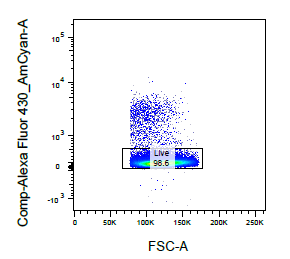 |  | 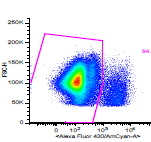 | 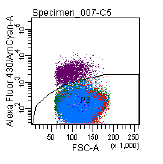 |
| 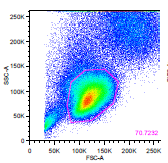 | 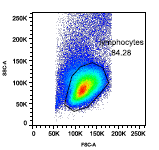 | 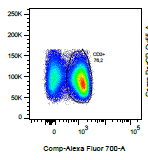 | 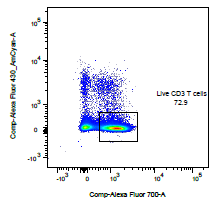 | 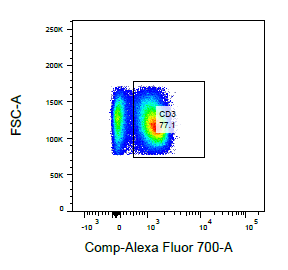 |  | 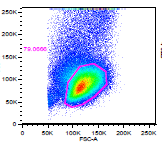 | 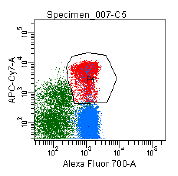 |
| 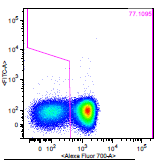 | 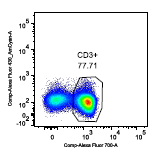 | 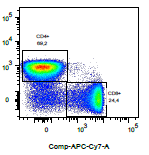 | 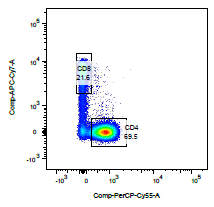 | 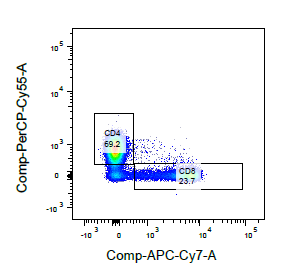 |  | 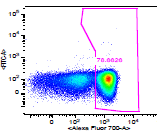 | 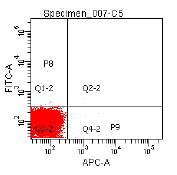 |
| 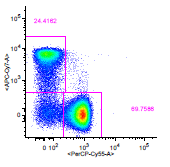 | 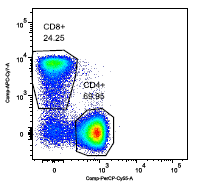 | 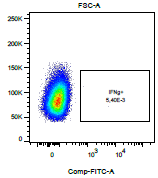 | 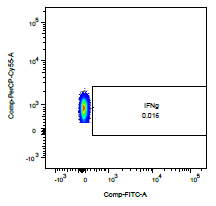 | 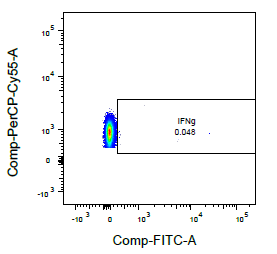 |  | 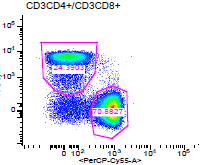 |  |
| 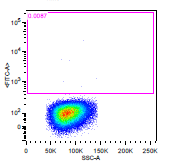 | 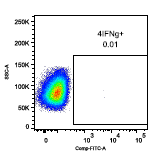 |  |  |  |  | 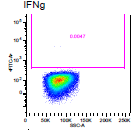 |  |
